# Polymers in Lithium–Sulfur Batteries

**DOI:** 10.1002/advs.202103798

**Published:** 2021-11-05

**Authors:** Qing Zhang, Qihua Huang, Shu‐Meng Hao, Shuyi Deng, Qiming He, Zhiqun Lin, Yingkui Yang

**Affiliations:** ^1^ Key Laboratory of Catalysis and Energy Materials Chemistry of Ministry of Education & Hubei Key Laboratory of Catalysis and Materials Science Hubei Engineering Technology Research Centre of Energy Polymer Materials South‐Central University for Nationalities Wuhan 430074 China; ^2^ School of Materials Science and Engineering Georgia Institute of Technology Atlanta GA 30332 USA

**Keywords:** binders, cathodes, electrolytes, lithium–sulfur batteries, polymers

## Abstract

Lithium–sulfur batteries (LSBs) hold great promise as one of the next‐generation power supplies for portable electronics and electric vehicles due to their ultrahigh energy density, cost effectiveness, and environmental benignity. However, their practical application has been impeded owing to the electronic insulation of sulfur and its intermediates, serious shuttle effect, large volume variation, and uncontrollable formation of lithium dendrites. Over the past decades, many pioneering strategies have been developed to address these issues via improving electrodes, electrolytes, separators and binders. Remarkably, polymers can be readily applied to all these aspects due to their structural designability, functional versatility, superior chemical stability and processability. Moreover, their lightweight and rich resource characteristics enable the production of LSBs with high‐volume energy density at low cost. Surprisingly, there have been few reviews on development of polymers in LSBs. Herein, breakthroughs and future perspectives of emerging polymers in LSBs are scrutinized. Significant attention is centered on recent implementation of polymers in each component of LSBs with an emphasis on intrinsic mechanisms underlying their specific functions. The review offers a comprehensive overview of state‐of‐the‐art polymers for LSBs, provides in‐depth insights into addressing key challenges, and affords important resources for researchers working on electrochemical energy systems.

## Graphical abstract

A comprehensive summary on multifunctional roles of emerging polymers in lithium–sulfur batteries in terms of cathodes, binders, interphases, separators, and electrolytes is presented rationally, to provide new insights into the rational development of polymers for addressing current challenges of lithium–sulfur batteries.

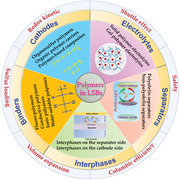

## 1 Introduction

The rapid economic/social development and ever‐increasing energy consumption have brought severe environmental pollution and energy crisis. Renewable energy technologies such as wind and solar energy outside of conventional fossil energy have effectively alleviated the current energy shortage. However, such energy sources are impossible to achieve sustainable supply due to their intermittence features of geographical environment and natural conditions.^[^ ^1^ ^]^ In response to this situation, electrochemical energy technologies particularly for rechargeable lithium‐ion batteries (LIBs) have received major attention and successfully predominated portable electronics over the past decades.^[^ ^2^, ^3^, ^4^ ^]^ Against the backdrop of the fast‐emerging electric vehicles (EVs) in recent years, commercial LIBs cannot longer fulfil the practical demand due to their limited energy density less than 260 Wh kg^−1^.^[^ ^5^ ^]^ The main bottleneck is the capacity limitation of intercalation‐type cathode materials such as LiFePO_4_ and LiCoO_2_.^[^ ^6^, ^7^ ^]^ Their capacities are infinitely close to the theoretical values without a large room to be further improved. Therefore, it is hard to significantly increase the energy density of current LIBs from the perspective of electrode materials. Exploring new battery configurations beyond LIBs is urgently required for the development of the next‐generation high energy batteries.

In this regard, lithium–sulfur batteries (LSBs) based on sulfur cathodes have aroused great interest in academia and communist industry due to their extremely high theoretical energy density (≈2600 Wh kg^−1^).^[^ ^8^, ^9^, ^10^, ^11^ ^]^ The high theoretical capacity (1675 mAh g^−1^) of elemental sulfur perfectly makes up for the shortage of its relatively low working voltage (2.2 V).^[^ ^12^ ^]^ In addition, the natural abundance and cost‐effectiveness of sulfur also endow LSBs the possibility for large‐scale application.^[^ ^13^ ^]^ In fact, the concept of LSBs has been proposed as early as 1960s, and the Li–S battery chemistry has also been systematically studied in the past few decades.^[^ ^7^, ^14^ ^]^ Unfortunately, the unsatisfactory cycle life has restricted the development of LSBs for a long time, resulting in the unsuccessful commercialization.^[^ ^15^, ^16^ ^]^ The notorious “shuttle effect” caused by the dissolution of lithium polysulfides (LiPSs) intermediates in organic electrolytes and their migration from the cathode to the anode are mainly responsible for the poor cycling stability.^[^ ^17^, ^18^ ^]^ Besides, the migration of dissolved LiPS may cause the self‐discharge even at the storage and rest state.^[^ ^19^ ^]^ Meanwhile, the intrinsic electronic insulation of sulfur (5 × 10^−30^ S cm^−1^) and discharged products of lithium sulfide (Li_2_S) (3 × 10^−7^ S cm^−1^) also lead to low sulfur utilization and poor rate capability.^[^ ^10^, ^20^ ^]^ In addition, the sulfur cathodes based on a conversion mechanism has a huge volume expansion (80%) during the discharge/charge processes, and this leads to the pulverization of active materials and shedding from the current collector.^[^ ^21^ ^]^ Of course, the safety issue caused by lithium dendrites in lithium metal anode is also ignored.^[^ ^22^, ^23^, ^24^ ^]^

To overcome the above challenges, fabricating sulfur composite cathodes to enhance electronic conductivity, accommodate volume change, and suppress shuttle effect are crucial. In the last years, most investigations on LSBs have mainly focused on the design and modification of sulfur cathodes.^[^ ^13^, ^14^, ^25^, ^26^, ^27^ ^]^ Since Ji et al.^[^ ^28^ ^]^ reported the carbon/sulfur composite cathode by using mesoporous carbon as the sulfur carrier in 2009, tremendous sulfur‐based composites cathodes with carbonaceous materials,^[^ ^29^ ^]^ metal compounds,^[^ ^30^ ^]^ and metal–organic frameworks^[^ ^31^ ^]^ as carriers have been recently demonstrated to address the aforementioned issues. The elaborated carbon architectures can not only promote the electronic conductivity of the sulfur cathode, but also realize spatial confinement of active materials and LiPS in nanopores. Nevertheless, the inert surfaces and nonpolar feature of most carbon carriers make them difficult to provide sufficient chemical binding sites to the supporting sulfur.^[^ ^32^ ^]^ The weak interaction based on physical adsorption is insufficient to inhibit the LiPS dissolution.^[^ ^33^ ^]^ Polar metal‐containing compounds are also used as sulfur carriers, which can chemically anchor LiPS due to their “sulfiphilic” surface. However, the incorporation of metallic compounds would reduce the energy density of the electrode. It is also difficult to achieve a high sulfur loading due to their insufficient adsorption sites.^[^ ^34^ ^]^ In addition to the fabrication of sulfur composite cathodes, the modification of binders and design of multifunctional separators,^[^ ^35^ ^]^ the introduction of interphases between the cathode and separator,^[^ ^36^ ^]^ and the design of solid electrolyte^[^ ^37^ ^]^ are also of great importance to suppress the shuttle effect, thereby synergistically improve the electrochemical performance of LSBs.

**Figure 1:**
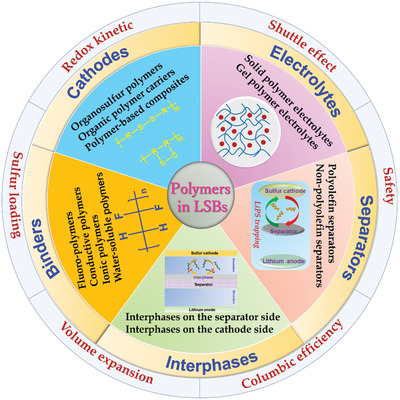
Multifunctional roles of polymers in addressing current challenges of lithium–sulfur batteries.

At present, polymers have received considerable attention in LSBs due to their rich abundance, lightweight, numerous molecular structures, and definable functional groups.^[^ ^38^, ^39^, ^40^, ^41^, ^42^, ^43^ ^]^ Polymers can not only function as elemental sulfur carriers, but also directly serve as the active cathode materials such as organosulfur polymers. Compared to nonpolar carbon carriers based on ordinary physical adsorption, the elaborated polymers can be rich in polar functional groups, thereby exhibiting abundant chemical binding sites and stronger affinity to LiPS. The shuttle effect can be remarkably inhibited by the covalent interaction between functional groups and LiPS. Furthermore, the flexible skeleton of polymers can effectively buffer the volume change of the sulfur cathode during the discharge/charge processes. Conductive polymers used as the sulfur carrier can further improve the electronic conductivity.^[^ ^44^ ^]^ Besides, compared to the melting impregnation synthesis of inorganic/sulfur composites, the sulfur‐mediated synthesis of sulfur‐containing polymers is usually performed under milder conditions, achieving a homogeneous distribution of sulfur. Moreover, polymers also play critical roles in binders, separators, and electrolytes instead of being merely limited to the cathode due to their excellent chemical stability, film‐forming ability, and processability as demonstrated in **Figure** **1**.^[^ ^36^, ^45^ ^]^

Considering the above important roles of polymers in LSBs, it is necessary and meaningful to thoroughly review the state‐of‐the‐art achievements of polymers in LSBs in terms of all aspects that affect performance. However, very few reviews have been focused on the advancement of functional polymers in LSBs.^[^ ^38^, ^39^ ^]^ This review systematically summarized the research progress of polymers in LSBs in terms of cathodes, binders, the interphases between the cathode and separators, separators, and electrolytes. Electrochemical‐redox sulfur‐containing polymers including various organosulfur polymers and polymer‐based carriers for sulfur have been emphatically discussed from the perspective of molecular engineering. An overview on multifunctional polymer binders, polymer interphases, separators, and electrolytes are also outlined. Future directions in developing polymer materials to tackle the critical challenges of LSBs are proposed finally.

## 2 Lithium–Sulfur Chemistries

**Figure 2:**
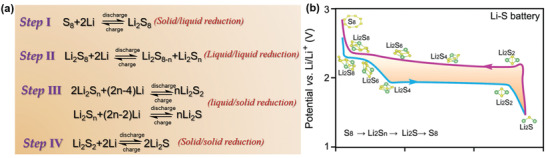
a) Multiple‐step redox reactions, and b) typical discharge/charge curves of LSBs. Reproduced with permission.^[^ ^46^ ^]^ Copyright 2021, Royal Society of Chemistry.

The discharge/charge processes of LSBs are achieved by the electrochemical cleavage and reformation of sulfur–sulfur bonds, associated with multiple‐step redox reactions and complex phase transfers of various LiPS, as shown in **Figure** **2**.^[^ ^46^, ^47^ ^]^ During the discharge process, elemental sulfur (S_8_) first combines with Li‐ions (step I) through a solid/liquid two‐phase reduction to form the dissolved Li_2_S_8_, which then reacts with Li‐ions to yield the long‐chain LiPS intermediates (Li_2_S*_n_*, 4 ≤ *n* ≤ 8) (step II) through a liquid/liquid reduction process. These two steps contribute to a theoretical capacity of 419 mAh g^−1^.^[^ ^48^ ^]^ The sequent reductions of S_8_ to S_6_^2−^ and S_4_^2−^ correspond to the discharge potential platform between 2.2 and 2.3 V (vs Li^+^/Li), leading to the decrease of sulfur chain length. The formed long‐chain Li_2_S*_n_* are easily soluble in organic electrolyte, which gradually separate from the cathode and diffuse into the electrolyte. With the continuous discharge process, long‐chain Li_2_S*_n_* are further reduced to short‐chain Li_2_S_2_ or Li_2_S with extremely low solubility in the electrolyte (step III). This liquid/solid two‐phase reduction corresponds to the second discharge platform between 1.9 and 2.1 V (vs Li^+^/Li) in the discharge/charge profiles, which contributes to the largest proportion (1256 mAh g^−1^) of theoretical capacity.^[^ ^49^ ^]^ Finally, the insulation and insolubility of Li_2_S_2_ and Li_2_S result in the large polarization and sluggish reaction kinetics at the last solid/solid reduction (step IV). During the charge process, Li_2_S_2_ and Li_2_S are gradually oxidized into LiPS intermediates, and finally transform to elemental sulfur. Throughout the discharge/charge process, the dissolution and diffusion of long‐chain LiPS is the main reason for the capacity decay and shuttle effect. Meanwhile, the migration of the dissolved LiPS from the electrolyte to the anode side may also result in the loss of active materials and corrosion of lithium metal anode, which would intensify the uneven deposition of lithium and cause serious pulverization.

## 3 Polymer‐Based Cathodes

As descripted in the introduction, the most effective strategy to improve the electrochemical performance of LSBs is designing high‐performance cathodes with high sulfur loading, enhanced electronic conductivity, and suppressed volume change and shuttle effect. The current reports about sulfur cathodes mainly focus on the design of sulfur carriers including nanocarbons and metal compounds. However, the seemingly perfect encapsulation of these carriers exhibits limited tapping ability to LiPS especially at a high sulfur content (>60 wt%), which would inevitably result in the partial escape of LiPS to a certain degree. In addition, the commonly employed melt impregnation synthesis approaches of most inorganic/sulfur composites are also complicated. In contrast, elaborated organic polymers with rich functional groups and tunable topological structures can be expected to simultaneously achieve high sulfur content with physical/chemical impregnation and robust LiPS trapping via strong covalent bonds. Polymer‐based sulfur cathodes mainly include electroactive sulfur‐containing polymer (organosulfur polymers) and polymer encapsulated sulfur cathodes.

### 3.1 Organosulfur Polymers

#### 3.1.1 Disulfide‐Linked Polymers

**Figure 3:**
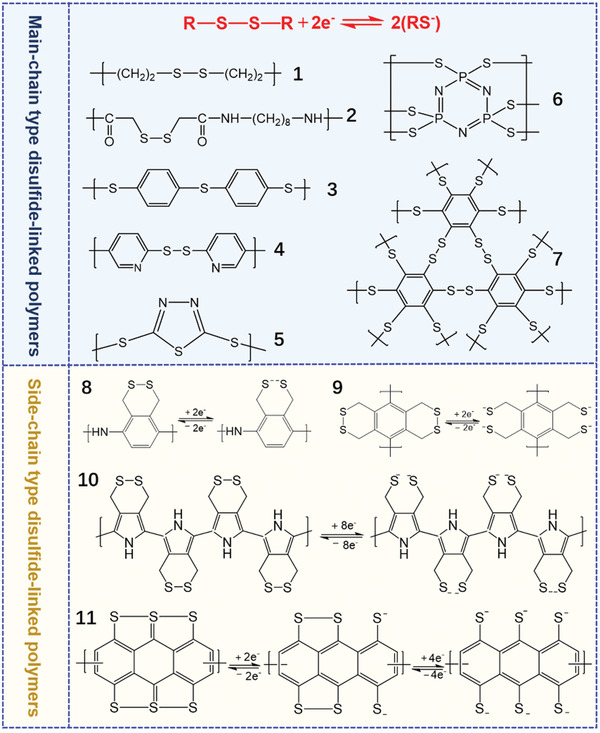
Molecular structures of main‐ and side‐chain type disulfide‐linked polymers (Nos. **1**,^[^ ^53^ ^]^ **2**,^[^ ^54^ ^]^ **3**,^[^ ^55^ ^]^ **4**,^[^ ^53^ ^]^ **5**,^[^ ^50^ ^]^ **6**,^[^ ^56^ ^]^ **7**,^[^ ^57^ ^]^ **8**,^[^ ^58^ ^]^ **9**,^[^ ^59^ ^]^ **10**,^[^ ^60^ ^]^ and **11** ^[^ ^61^ ^]^).

Organosulfur polymers can directly serve as the electroactive cathode material due to the presence of sulfur chains in the polymer backbone. Sulfur‐containing polymers with disulfide bonds (S—S) are the most representative one, which have been reported as early as 1990s.^[^ ^50^, ^51^ ^]^ The discharge/charge process of disulfide‐linked polymers is achieved through the cleavage and reconstruction of S—S bonds (S—S + 2e^−^ ⇌ 2S^−^), accompanied by the reversible lithiation/delithiation process. It has been proved that well‐designed organosulfur polymers can be expected to realize the capacity range of 360–580 mAh g^−1^ with the energy density of 720–1240 Wh kg^−1^, which provides a promising alternative to elemental sulfur cathode.^[^ ^52^ ^]^ Depending on the position of disulfide bonds in the polymer backbone, the current reported disulfide‐linked polymers can be classified into main‐chain and side‐chain types as shown in **Figure** **3**.

Main‐chain type disulfide‐linked polymers is the first kind of electroactive organosulfur polymers served as LSB cathodes, which mainly consist of organic moieties bonded with disulfide units as linkers in the main chain.^[^ ^50^, ^51^, ^62^ ^]^ Tsutsumi et al.^[^ ^53^, ^54^ ^]^ have early synthesized a series of linear polyamides (Nos. **1** and **2**) linked by disulfide units through the interfacial polymerization and investigated their application in lithium secondary batteries. Unfortunately, these polymers all exhibit drastic capacity decay in organic electrolyte in a few cycles (10 cycles) when directly served as the cathodes. The capacity loss is mainly ascribed to the fact that the cleavage of disulfide bonds during discharge process results in the irreversible break of the linear‐type polymer chain. Besides, most main‐chain polymers with limited disulfide units (Nos. **3**–**5**) possess low capacities (<250 mAh g^−1^) far below the theoretical capacity of elemental sulfur, which severely restrict their application.^[^ ^55^ ^]^ In order to increase the sulfur content and strengthen the stability of the polymer skeleton, crosslinked disulfide‐bonded polymers with high sulfur content were recently synthesized through simple polycondensation reactions of monomers and elemental sulfur (S) (Nos. **6** and **7**).^[^ ^56^, ^63^ ^]^ Preefer et al.^[^ ^57^ ^]^ recently designed a crosslinked organosulfur polymer containing rich disulfide bonds (No. **7**), which maximizes the ratio of S–S units to electrochemically inactive components with a 1:1 ratio of S:C in the polymer backbone. The rich disulfide bonds endow this polymer a high theoretical capacity (609 mAh g^−1^). Ex situ Raman spectroscopy investigation confirms that the redox mechanism of this crosslinked polymer is the break of S–S to form S–Li thiolate species during discharge and reforming of S—S bond upon charge process. It is worth mentioning that the cleavage of S—S bonds would not result in the damage of the stable crosslinked backbone. Therefore, the crosslinked polymer can prevent the dissolution of LiPS through the physical/chemical confinement and maintain structural stability as much as possible. When used as LSBs cathode, it maintains a high capacity retention up to 98% after 200 cycles, suggesting a remarkable promoted cycling stability compared to the previously reported linear disulfide‐linked polymers. Especially, it gains almost 100% of Coulombic efficiency through the whole cycles, suggesting that the LiPS shuttle has been remarkably prevented. Nevertheless, this polymer delivers a relatively low practical capacity of 159 mAh g^−1^ at C/10, due to the incomplete conversion of all S—S bonds during cycling.

As mentioned above, the repeated cleavage and reforming of disulfide bonds existing in the main chain would inevitably result in the irreversible structure variation of polymer backbone in the long‐term cycles. The dissolution of the depolymerized monomers in electrolyte also further leads to the loss of active materials.^[^ ^51^, ^64^ ^]^ In this regard, side‐chain type organosulfur polymers have been investigated (Nos. **8**–**11**),^[^ ^60^, ^61^, ^65^ ^]^ because the electrochemical reaction of disulfide bond located on the side chain would not affect the polymer backbone, thus enabling better structural stability upon cycling. Deng et al.^[^ ^59^ ^]^ have early explored the application of benzene‐based polyorganodisulfide derivative (PDTTA) (No. **9**) in LSBs. PDTTA possess a high theoretic specific capacity of 471 mAh g^−1^ due to the presence of two disulfide bonds. The remarkable advantage of this polymer is that the reversible cleavage/recombination of S—S bonds occur in each monomer unit, which would not cause damage to the polymer backbone. The main chain of PDTTA can be well maintained during repeated discharge/charge processes. Besides, the redox process of S—S bonds can be enhanced by the intramolecular electrocatalysis of polyphenyl units. Therefore, PDTTA delivers a high initial capacity of 422 mAh g^−1^ and maintains a stable capacity of 170 mAh g^−1^ after 44 cycles at 30 mA g^−1^. Aniline‐based polyorganodisulfide (PDTAn) (No. **8**) containing one S—S bond on the side chain has also been explored as LSBs cathode.^[^ ^58^ ^]^ The similar molecular structure to conducting polyaniline (PANI) not only contributes to high electronic conductivity with fast redox kinetic, but also endows a high theoretical capacity (≈370 mAh g^−1^). Meanwhile, the intramolecular redox action of S—S bond does not result in the depolymerization of polyaniline chain, thereby endows enhanced reversibility. It shows an initial discharge capacity of 225 mAh g^−1^ at 10 mA g^−1^ and keeps the Coulombic efficiency of more than 80% in the following cycles. Although the side‐chain‐type organosulfur polymers exhibited enhanced cycling stability compared to the main‐chain types, the low specific capacity due to the limit disulfide bonds and large amounts of inactive components in the molecular backbone as well as poor discharge capability at large rates still restrict their practical application. The future exploration about the side‐chain‐type organosulfur polymers should be focused on achieving the compromise between the capacity and stability by regulating the ratio of disulfide bonds and inactive units. In addition, the electronic conductivity of organosulfur polymers also needs to be enhanced to promote the sulfur utilization and rate capability. Designing crosslinked conjugated backbone containing rich disulfide bonds through appropriate sulfur‐mediated synthesis is expected to simultaneously achieve high capacity, high sulfur utilization, and satisfactory cycle life.

#### 3.1.2 Sulfurized Polymers

**Figure 4:**
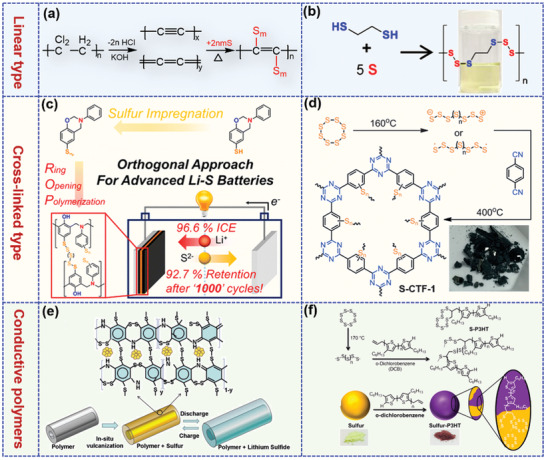
Sulfurized polymers: a) The synthetic route of the carbyne polysulfide. Reproduced with permission.^[^ ^68^ ^]^ Copyright 2013, Royal Society of Chemistry. b) The synthetic route of polyethylene hexasulfide (PEHS). Reproduced with permission.^[^ ^69^ ^]^ Copyright 2019, American Chemical Society. c) The synthetic route and electrochemical performance of sulfur‐embedded polybenzoxazine (S‐BOP). Reproduced with permission.^[^ ^70^ ^]^ Copyright 2016, American Chemical Society. d) The synthetic route and an optical image of sulfurized covalent triazine frameworks (S‐CTFs). Reproduced with permission.^[^ ^71^ ^]^ Copyright 2016, Wiley‐VCH. e) The synthetic and discharge/charge process schematic of SPANI‐NT/S. Reproduced with permission.^[^ ^72^ ^]^ Copyright 2012, Wiley‐VCH. f) The synthetic route and proposed microstructure of sulfurized polythiophene (S‐P3HT). Reproduced with permission.^[^ ^73^ ^]^ Copyright 2015, American Chemical Society.

The simple electroactive disulfide‐linked polymers can only contribute limited capacity due to the low sulfur content, which does not exceed 50 wt% even in crosslinked polymer backbones with rich disulfide bonds. As a consequence, the rational design of organosulfur polymers with inherently high sulfur content and robust structural stability is essential to promote the practical capacity and thus realize their practical application.^[^ ^66^ ^]^ It has been well known that stable elemental sulfur consists of S_8_ molecules, which would break into various chain‐like free radical (•Sn•) with diradical chain ends when heating the sulfur to high temperature (159–444 °C).^[^ ^49^ ^]^ Therefore, adjusting sulfur chains (R–S*_n_*–R, *n* > 2) in the polymers through facile copolymerization of monomers and the highly active •Sn• free radicals has been the simplest strategy to obtain sulfurized polymers with high sulfur content (up to 80 wt%), namely, vulcanization process.^[^ ^38^, ^49^ ^]^ More importantly, the shuttle effect can be also minimized by controlling the length of the LiPS chain.^[^ ^67^ ^]^ By designing copolymerized monomers with different functional groups, numerous sulfurized polymers have been investigated as cathode materials for LSBs as displayed in **Figure** **4**.

As early as in 2013, Wang and co‐workers^[^ ^68^ ^]^ reported a carbyne polysulfide by the coheating process of carbyne monomer and elemental sulfur (Figure 4a). The sulfur molecules S*_m_* (1 ≤ *m* ≤ 4) are chemically linked to the carbon of ruptured C═C and C≡C bonds, thus realizing ≈54.1 wt% sulfur content. When used as LSBs cathode, the carbyne polysulfide shows a stable capacity of 960 mAh g^−1^ at 0.1C even after 200 cycles. The outstanding cycling stability is mainly due to the fact that the small S*_m_* units can directly combine Li‐ions to form Li_2_S/Li_2_S_2_, which avoids the generation of soluble long‐chain LiPS. The carbyne polysulfide thus demonstrates the solid‐phase redox process with a single discharge plateau. When the rate increases to 1C, the capacity can be maintained about 705 mAh g^−1^, indicating a superior rate capability, which can be explained by the promoted electronic conductivity due to the conjugated carbon skeleton of carbyne structure. Recently, Bhargav et al.^[^ ^69^ ^]^ designed a sulfur‐rich polymer (polyethylene hexasulfide, PEHS) through a facile condensation reaction of elemental sulfur and 1,2‐ethanedithiol containing thiol linkage group (—SH) (Figure 4b). Herein, six linear sulfurs are connected by the inserted ethylene groups, leading to a high sulfur content (up to 87 wt%). PEHS thus gives a theoretical capacity of 1217 mAh g^−1^ based on a 10‐electron transfer. Meanwhile, the choice of —SH linkage group with low molecular weight also allows for a precise control of the LiPS chain length. Therefore, PEHS delivers a large capacity up to 1108 mAh g^−1^ at C/20, and maintains about 774 mAh g^−1^ when the rate increases to 1C. It also exhibits an excellent cycling stability with a capacity retention up to 71% after 350 cycles at 1C. More importantly, the Coulomb efficiency can be kept above 99%, indicating a significantly inhibited shuttle effect. The superior LiPS shuttle prevention is mainly attributed to the minimized formation of long‐chain LiPS during discharge process, which plagues most conventional sulfur cathodes.

Although the extended S–S units in the polymer chains can increase the capacity, the inevitable formation of long‐chain LiPS intermediates during discharge process still would result in the capacity decay. Therefore, improving the chemical impregnation of sulfur and strengthening the chemical LiPS trapping ability via strong covalent bond is still of great importance for organosulfur polymers. On this account, side‐chain type and crosslinked sulfurized polymers including organic frameworks are employed to increase the sulfur content while ensuring the cycling stability. Je et al.^[^ ^70^ ^]^ designed a sulfur‐impregnated benzoxazine polymer (S‐BOP) through the ring‐opening polymerization of thiol‐functionalized benzoxazine monomer (Figure 4c). The thiol moieties provide the binding sites for sulfur impregnation to form short sulfur side‐chains (S*_n_*, *n* = 2–6) between the neighboring polymer backbones. The resulted S‐BOP shows a high sulfur content of 72 wt% with homogeneous distribution. Due to the covalent attachment of sulfur in BOP backbone and the reversible reaction of C—S and S—S bonds, S‐BOP cathode demonstrates an outstanding cycling stability with a high capacity retention of 92.7% even after 1000 cycles. Besides, the favorable interaction between the LiPS and heteroatoms in the polymer backbone as well as evenly distributed sulfur can effectively impede the dissolution of LiPS. Meanwhile, the Coulombic efficiency can maintain up to 96.6% along the whole cycles, suggesting the remarkably suppressed shuttle effect. Ordered framework structures were also employed to chemically attach sulfur with homogeneous distribution due to their porous characteristic, large surface areas, and moderate ionic/electronic conductivity.^[^ ^74^ ^]^ Talapaneni et al.^[^ ^71^ ^]^ synthesized a sulfurized covalent triazine frameworks (S‐CTFs) through an in situ vulcanization process without any catalyst and solvent (Figure 4d). The formed framework structure not only enables chemical impregnation of sulfur via covalent attachment, but also realizes a high sulfur content of 62 wt% with regular distribution. When served as LSBs cathode, the S‐CTF electrode shows a robust cycling stability with 85.8% capacity retention after 300 cycles at 1C. Even cycled at 2C, the capacity retention still maintains above 80% for prolonged 300 cycles. It is noteworthy that the initial Coulombic efficiency achieves up to 94.4% and rises quickly to above 99.5% after 5 cycles at both 1C and 2C. The stable cycle life and high Coulombic efficiency are mainly attributed to that the regular sulfur distribution within the micropores and the strong C–S covalent links together prevent the dissolution of LiPS. Besides, N atoms within triazine units can stabilize LiPS via the chemical interaction with Li‐ions. The microporous structure of CTF also provides large void spaces for buffering the volume change. In addition, the triazine framework can facilitate fast ion/electron transfer due to its order micropores, thereafter contributing to superior rate capability and high sulfur utilization.

Most sulfurized polymers exhibit electrical insulation, resulting in slow reaction kinetics and poor rate capability. Coupling sulfur into the backbone of conductive polymers such as polypyrrole (PPy), polyaniline (PANI), and polythiophene (PTh) through a vulcanization process is usually expected to obtain sulfurized polymers with high electronic conductivity.^[^ ^75^ ^]^ In view of this, Xiao et al.^[^ ^72^ ^]^ designed a sulfur‐polyaniline (SPANI‐NT/S) with a high sulfur content of 62 wt% through an in situ vulcanization method (Figure 4e). Due to the inter‐ and intrachain disulfide bonds interconnected polymer backbone, the sulfur is both physically adsorbed within the 3D network and chemically bonded to molecular chains. Benefiting from the high electronic conductivity of polyaniline skeleton and the significantly inhibited shuttle effect by physically/chemically anchoring, SPANI‐NT/S delivers a stable capacity of 837 mAh g^−1^ at 0.1C after 100 cycles. Even at a large rate of 1C, the capacity can be still maintained above 400 mAh g^−1^ after 500 cycles, suggesting a superior rate capability and cycling stability. Polythiophene with allyl end‐groups has been also applied to copolymerize with sulfur to form sulfurized polythiophene (S‐P3HT) (Figure 4f).^[^ ^73^ ^]^ Sulfur atoms are covalently linked to allyl‐terminated semiconductive backbone, remarkably promoting charge transfer and decreasing the loss of active materials due to the strong chemical interaction between polythiophene and LiPS. However, the limited allyl terminal groups of P3HT can only covalently bond a small proportion of sulfur. The noncovalently bonded sulfur inevitably causes a certain capacity loss. In order to address this problem, Zeng et al.^[^ ^76^ ^]^ further reported the random copolymerization of 3‐butylthiophene and sulfur (P3BT) through simple radical polymerization. Numerous covalently bonded sulfur in the main chain can substantially suppress the dissolution of LiPS due to the strong chemical confinement. Besides, the crosslinked network also provides effective physical confinement to LiPS. By capping the as‐prepared P3BT in conductive poly(3,4‐ethylene‐dioxythiophene):poly(styrenesulfonate) (PEDOT:PSS) thin film, the hybrid exhibits an initial discharge capacity up to 1362 mAh g^−1^ at 0.1C. Unsurprisingly, the hybrids demonstrate a superior stability with a decay rate of only 0.053% per cycle after 500 cycles at 1C.

In spite of the significantly increased sulfur content of sulfurized polymers compared to disulfide‐linked polymers, future work should concentrate on controlling the length of sulfur chains to avoid the dissolution of generated high‐order LiPS. Meanwhile, the incorporation of conjugated backbone should be considered to accelerate the electron transport and promote the sulfur utilization. In addition, the lithium storage mechanism and structural evolution need to be deeply investigated to provide more powerful guides for the design of sulfurized polymers. Nevertheless, the above reports indicate that the design of sulfurized polymers through the facile vulcanization process would open up a new path to build organosulfur polymers with high capacity, stable cycle stability, and superior rate capability.

#### 3.1.3 Sulfurized Polyacrylonitrile

Among various sulfurized polymers, sulfurized polyacrylonitrile (S‐cPAN) has been regarded as the most promising cathode for LSBs due to the remarkably improved stability compared to other sulfur‐containing polymers.^[^ ^77^, ^78^, ^79^, ^80^ ^]^ Different from the dramatic capacity decay existed in most sulfur cathodes caused by the serious shuttle effect, the optimized S‐cPAN with high sulfur utilization (85%) can achieve a superior capacity retention over 90% even after 2000 cycles.^[^ ^81^ ^]^ S‐cPAN is generally synthesized through the dehydrogenation of polyacrylonitrile (PAN) and cyclization during high temperature vulcanization process. The sublimed sulfur can react with the dehydrogenated PAN to form heterocyclic polymer (cPAN) due to the formation of disulfide bonds accompanied by the release of H_2_S. Up to now, the detail molecular structure of S‐cPAN and the embedding form of sulfur in cyclic PAN backbone have not yet fully unveiled.^[^ ^82^ ^]^ Sulfur has been considered to exist in nanodispersed elemental form due to the undetected C—S bonds in Fourier‐transform infrared spectroscopy (FT‐IR) in the early reports.^[^ ^77^, ^83^ ^]^ However, some reports have confirmed that sulfur was covalently anchored in PAN backbones in the form of short —S*_x_*— chains (C—S bonds).^[^ ^84^ ^]^ The essential reason for the distinctive cycling stability of S‐cPAN compared to other sulfur cathodes has been also widely discussed in the past few years.^[^ ^85^, ^86^ ^]^ The most common viewpoint is the possible “solid–solid redox mechanism” without the formation of long‐chain LiPS during discharge process. The covalent C—S bonds on cPAN backbones can completely eliminate the shuttle effect.^[^ ^87^ ^]^

**Figure 5:**
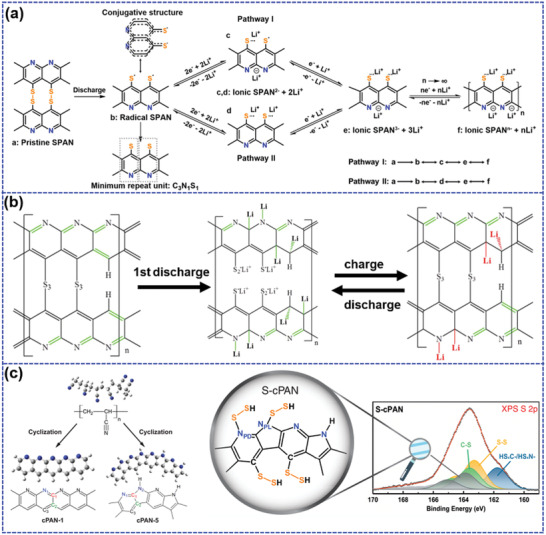
a) The proposed molecular structure and reaction pathway of S‐cPAN. Reproduced with permission.^[^ ^85^ ^]^ Copyright 2018, American Chemical Society. b) The possible molecular configuration and redox mechanism of S‐cPAN. Reproduced with permission.^[^ ^88^ ^]^ Copyright 2018, Elsevier. c) Structure diagrams of cPAN with different nitrogen configurations and the proposed molecular of S‐cPAN. Reproduced with permission.^[^ ^89^ ^]^ Copyright 2021, American Chemical Society.

Wang et al.^[^ ^85^ ^]^ proposed a highly possible molecular configuration and raised a new understanding on the Li‐storage mechanism of S‐cPAN through the combination of experiments and theoretical calculations (**Figure** **5**a). They found that Li‐ions can reversibly react with negative sulfur and nitrogen atoms via forming ion‐coordination bonds. During the initial discharge process, the cleavage of S—S bonds results in the formation of thiyl radical, which then forms a conjugative structure due to electron delocalization on the pyridine backbone. In the following lithiation process, Li‐ions can accommodate on the negative S and N sites of conjugative structure through two pathways to form lithiated S‐cPAN. Herein, pathway I is considered to be preferable due to the lowest potential energy. During the charge process, the formed ionic S‐cPAN can reversibly transform to radical S‐cPAN. Therefore, the second discharge process starts from the radical S‐cPAN rather than the cleavage of the S–S, which well explains the distinction between the initial and second discharge curve. The redox process of radical S‐cPAN with fast electron transfer successfully eliminates the formation of soluble long‐chain LiPS during discharge process, which enables superior cycling life and rate capability. Jin et al.^[^ ^88^ ^]^ also reported similar molecular structure of S‐cPAN (Figure 5b). The sulfur radicals formed under high temperature vulcanization process chemically combine the cyclized polypyridine rings via S—C bonds. The neighboring polypyridine rings are connected by S*_x_* (2 ≤ *x* ≤ 4) chains through the generation of S—S bonds. Herein, they found that the conjugated double‐bond (C═N and C═C) can also storage Li‐ions instead of the mere Li‐storage on C–S*_x_* groups. During the initial discharge process, the transform from C—S*_x_* bonds to C–S–Li and the reaction of C═N and C═C groups with Li‐ions occur simultaneously. However, the formed Li—C—N—Li and Li—C—C—Li can only partially convert to C═N and C═C groups in the charge process, thus resulting in the irreversible capacity loss of initial cycle. It is noted that the residue Li‐ions in S‐cPAN backbone after the first charge can help promote the electronic conductivity, hence leading to the higher discharge plateau with lower polarization during the second discharge process.

Recently, Huang et al.^[^ ^89^, ^90^ ^]^ proposed another probable molecular structure of S‐cPAN with the coexistence of pyridinic and pyrrolic nitrogen (NPD and NPL) (Figure 5c). They first unraveled the presence of a significant amount of NPL in S‐cPAN, which is different from the predominant NPD in most previous reports.^[^ ^85^, ^91^ ^]^ Therefore, the proposed molecular configuration of cPAN (cPAN‐5) is composed of both six‐ and five‐membered rings containing NPD (N1) and NPL (N2) atoms. Theoretical calculation displays that S_2_ molecules exhibit the strongest adsorption ability to N atoms, consequently resulting in the formation of N—S bonds in addition to the formation of generally reported C—S bonds during vulcanization process.

Nevertheless, the exact molecular structure and redox mechanism of S‐cPAN still remain debatable. Future work should be continuously focused on addressing these two critical matters through advanced in situ characterization technologies combined with theoretical calculation. To be noticed, the current reported S‐cPAN generally possess a relatively low sulfur content, leading to lower capacity than other sulfur‐based cathodes. Therefore, promoting the sulfur content without the sacrifice of cycle life still remains a challenge. Meanwhile, the enhancement of electronic conductivity through hybridization with conductive carbon is also essential for further development of Li/S‐cPAN batteries. Besides, S‐cPAN demonstrates quite different electrochemical behaviors in different electrolytes such as carbonate‐ and ether‐based electrolytes, which also needs to be investigated in‐depth.^[^ ^92^ ^]^

#### 3.1.4 Inversely Vulcanized Copolymers

**Figure 6:**
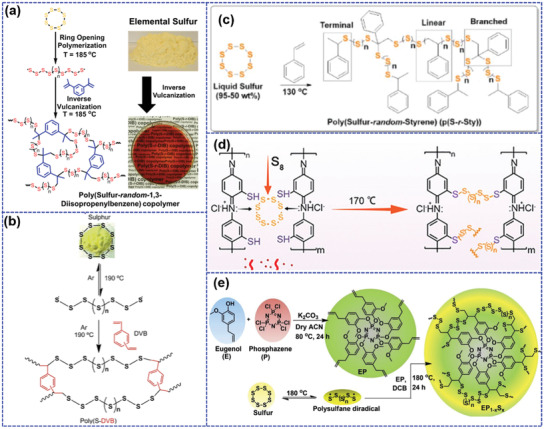
Inversely vulcanized copolymers: a) The synthetic scheme of poly(S‐r‐DIB). Reproduced with permission.^[^ ^96^ ^]^ Copyright 2014, American Chemical Society. b) The synthetic scheme of poly(S‐DVB). Reproduced with permission.^[^ ^94^ ^]^ Copyright 2016, Elsevier. c) The synthetic scheme and proposed structure of poly(S‐r‐Sty). Reproduced with permission.^[^ ^99^ ^]^ Copyright 2016, Wiley Periodicals, Inc. d) The synthesis route of cp(S‐PMAT). Reproduced with permission.^[^ ^101^ ^]^ Copyright 2017, Wiley‐VCH. e) The synthetic strategy of EPS copolymer. Reproduced with permission.^[^ ^102^ ^]^ Copyright 2020, Elsevier.

Although the vulcanization process based on the direct copolymerization of •Sn• diradical ends and monomers is considered to be the simplest strategy to form sulfur‐containing polymers, the traditional vulcanization process generally forms polymers like synthetic rubber with a small portion of sulfur content. Besides, the linear polysulfane obtained from the ring‐opening polymerization of S_8_ molecules may depolymerize back to the ring form when decreasing the temperature. In view of this, copolymerizing large amounts of sulfur with specific linkers such as diene monomers is generally employed to prevent the depolymerization of polymeric sulfur, namely, inverse vulcanization.^[^ ^49^ ^]^ Consequently, the higher sulfur content of the inversely vulcanized copolymers can reach up to 90 wt%. The most famous linker is 1,3‐diisopropenylbenzene (DIB), which has been widely investigated as crosslinking agent due to its thermodynamic compatibility with the molten sulfur.^[^ ^93^, ^94^ ^]^ Chung et al.^[^ ^95^ ^]^ synthesized a sulfur‐rich copolymer (poly(S‐r‐DIB)) with a high sulfur content of 90 wt% through the copolymerization of elemental sulfur and DIB monomer. Then they systematically investigated the composition effects on the electrochemical performance of poly(S‐r‐DIB) (**Figure** **6**a).^[^ ^96^ ^]^ The content of electroactive S–S units can be easily tuned by controlling the mass of DIB upon inverse vulcanization process. Poly(S‐r‐DIB) with 10 wt% DIB performs the optimized electrochemical performance with a stable capacity of 1005 mAh g^−1^ after 100 cycles at C/10. The capacity can be maintained at about 635 mAh g^−1^ ever after 500 cycles. The excellent cycling stability can be ascribed to that organosulfur DIB units generated during discharge process can function as “plasticizers” in insoluble LiPS (Li_2_S_3_/Li_2_S_2_), which avoids the capacity loss caused by the irreversible deposition of these sulfide phases. Then they further introduced polythiophene segments into the side chains of poly(S‐r‐DIB) backbone by using inverse vulcanization method.^[^ ^97^ ^]^ As expected, the resulted copolymer exhibits enhanced electronic conductivity due to the presence of conductive polythiophene units compared to poly(S‐r‐DIB).

Although DIB can offer controllable sulfur contents with homogeneous sulfur distribution in the crosslinked frameworks, DIB is an expensive crosslinker agent and shows slow copolymerization kinetics, which is not suitable for large‐scale synthesis of inversely vulcanized polymers.^[^ ^98^ ^]^ In view of this, Gomez et al.^[^ ^94^ ^]^ employed a cheaper divinylbenzene (DVB) as crosslinker to synthesize sulfur‐DVB copolymers (poly(S‐DVB)) through inverse vulcanization method (Figure 6b). The high reactivity of DVB monomer enables remarkably shortened reaction time (5 min) compared to that (1 and 2 h) of most reported inverse vulcanizations. Poly(S‐DVB) with 20 wt% DVB shows an initial capacity of 950 mAh g^−1^ at C/4 and keeps a reversible capacity of 700 mAh g^−1^ after 500 cycles, which can be comparable to conventional carbon/sulfur composites. Zhang et al.^[^ ^99^ ^]^ further employed styrene with lower cost as comonomer to prepare poly(sulfur‐random‐styrene) (poly(S‐r‐Sty) (Figure 6c). The application of styrene comonomer with widespread industrial use is expected to achieve large‐scale synthesis of high sulfur content copolymer. When served as LSBs cathode, poly(S‐r‐Sty) with 85 wt% sulfur delivers an initial capacity of 1048 mAh g^−1^ and exhibits a similar cycling stability to poly(S‐r‐DIB) cathode. A stable capacity of 485 mAh g^−1^ can be preserved even after a long‐term 1000 cycles at C/5. The enhanced cycle lifetime is also attributed to the “plasticization effect.” Besides, the copolymer network also functions as the mechanical stabilizer to prevent the possible mechanical damage upon cycles.

The above copolymers can enable high sulfur content with enhanced cycling stability by selecting appropriate crosslinker. However, most of them are insulator with low conductivity, which is not beneficial for the improvement of rate capability. Therefore, conductive backbones are recently used to copolymerize with element sulfur to overcome the above shortcoming.^[^ ^100^ ^]^ Zeng et al.^[^ ^101^ ^]^ synthesized a copolymer (cp(S‐PMAT)) through the copolymerization of conducting polymer poly(*m*‐aminothiophenol) (PMAT) with abundant thiol groups and elemental sulfur via inverse vulcanization (Figure 6d). The resulted cp(S‐PMAT) contains the crosslinked sulfur of ≈80 wt% content and inherits the conductive backbone of PMAT. It delivers a reversible capacity of 1240 mAh g^−1^ at 1C and maintains a high capacity of 600 mAh g^−1^ even at a large rate of 5C, suggesting a superior rate capability. The superb rate performance can be attributed to the continuous electron transport pathway provided by the PMAT segments. Meanwhile, cp(S‐PMAT) also performs an excellent cycling stability with the capacity decay of only 0.040% during 1000 cycles at 2C, which can be explained by the fact that the chemical bonds between sulfur and the thiol groups significantly inhibit diffusion of LiPS.

Designing crosslinkers can also address the security risks of LSBs caused by the intrinsic flammability of elemental sulfur. Monisha et al.^[^ ^102^ ^]^ innovatively proposed a flame‐inhibiting organosulfur copolymer (EPS) by employing eugenol phosphazene (EP) as crosslinker via inverse vulcanization process (Figure 6e). The flame retardant EP units endows EPS intrinsic fire‐retardant characteristics, which avoids the potential safety risks caused by the inherent flammable sulfur. The formed framework can effectively adsorb LiPS through physical confinement and chemical bonding simultaneously. The covalently tethered phosphazene framework can bear the volume expansion during the discharge/charge process. Therefore, the EPS with 83 wt% covalently linked sulfur cathode shows a large capacity 780 mAh g^−1^ at 0.2C and maintains a capacity retention of 60.3% after 200 cycles at 0.5C. More importantly, the burning test shows that EP/S ratio in the polymer backbone plays a critical role in flammable characteristics, which provides a new sight for developing safer and nonflammable LSBs.

As mentioned above, the inverse‐vulcanized polymers generally demonstrate the better electrochemical performance compared to the sulfurized polymers. The inverse vulcanization process directly uses elemental sulfur in the absence of other solvents and catalysts, and seems to be more readily than vulcanization process. However, the inescapable shuttle needs to be further optimized by macromolecular engineering of using multifunctional crosslinkers for the inverse‐vulcanized polymers. The copolymerization of sulfur and crosslinkers is also improved to realize the large‐scale synthesis of organosulfur polymers with high sulfur content.

### 3.2 Organic Polymer Carriers for Sulfur

#### 3.2.1 Conductive Polymers

**Figure 7:**
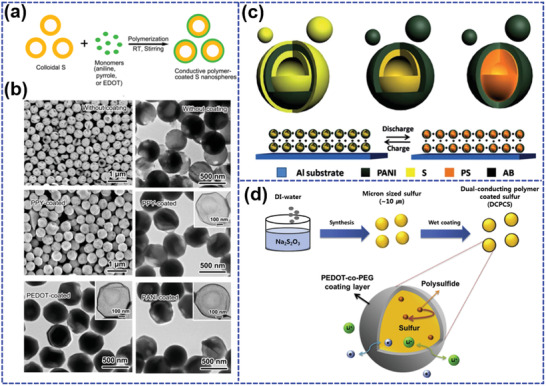
a) The fabrication process and b) morphologies of PPY‐S, PANI‐S, and PEDOT‐S. Reproduced with permission.^[^ ^106^ ^]^ Copyright 2013, American Chemical Society. c) The schematic of PANI‐S electrode during discharge/charge process. Reproduced with permission.^[^ ^107^ ^]^ Copyright 2014, Royal Society of Chemistry. d) The fabrication schematic of PEDOT‐*co*‐PEG coated sulfur composite. Reproduced with permission.^[^ ^108^ ^]^ Copyright 2018, Elsevier.

Traditional carbon‐based matrices as sulfur carriers can physically confine LiPS and promote the electronic conductivity of the cathodes. However, the poor binding ability between inert surface of carbon materials and polar Li*_x_* S (0 < *x* ≤ 2) species generally result in the detachment of Li*_x_* S from the carbon surface, thus leading to the loss of electrical contact.^[^ ^103^ ^]^ In this regard, conductive polymers with high electronic conductivity and polarity have been considered to be promising alternative sulfur carriers.^[^ ^44^, ^104^ ^]^ Building core–shell structure has been proved to be one of the most promising approaches to simultaneously promote the electronic conductivity and suppress the dissolution of LiPS.^[^ ^105^ ^]^ Li et al.^[^ ^106^ ^]^ employed typical conductive polymers PPY, PANI, and PEDOT as surface coating layers of monodisperse hollow sulfur nanospheres to synthesize PPY‐S, PANI‐S, and PEDOT‐S with core–shell structures through a facile aqueous polymerization process (**Figure** **7**a). Three composite cathodes demonstrate similar coating thicknesses of about 20 nm and approximate sulfur contents of ≈74 wt% (PANI), 77 wt% (PPY), and 78 wt% (PEDOT) (Figure 7b). The electrochemical impedance spectroscopy (EIS) shows that the conductivity decreases in the order of PEDOT > PPY > PANI. PEDOT‐S thus demonstrates the best rate capability with the capacity of 624 mAh g^−1^ at 4C, larger than those of PANI‐S (329 mAh g^−1^) and PPY‐S (440 mAh g^−1^). The ab initio simulations reveal that PEDOT exhibits a much stronger binding energy with lithium atom of Li–S• than PPY and PANI. Therefore, PEDOT‐S cathode shows the best cycling stability with 86% capacity retention after 500 cycles at C/2, larger than those of PANI‐S (65%) and PPY‐S (74%). The improved cycling stability is attributed to the physical confinement of Li*_x_* S (0 < *x* ≤ 2) within the coating layers and the stronger chemical bonding between the polymers and Li*_x_* S.

Ma et al.^[^ ^107^ ^]^ designed a PANI hollow spheres served sulfur reservoirs (PANI‐S) through vapor phase infusion method (Figure 7c). Sulfur is both uniformly adsorbed on the outer surface and inner of as‐prepared PANI hollow spheres. Although the sulfur on the surface may partially dissolve in the electrolyte in the initial few cycles, the loss of sulfur in the inner can be effectively inhibited by the protection of hollow PANI. In addition, the chemical bonding (S—C bonds) between PANI and sulfur can suppress the shuttle effect by preventing the dissolution and migration of polysulfide. Meanwhile, the void space in the hollow sphere can buffer the volume expansion of sulfur nanoparticles during discharge/charge process. Therefore, PANI‐S cathode demonstrates a superior long‐term cycling stability with the capacity up to 602 mAh g^−1^ even after 1000 cycles at 0.5C. Besides, PANI‐S also demonstrates an excellent rate capability with a high capacity of 785.7 mAh g^−1^ at a large rate of 5C, due to the enhanced electronic conductivity.

Although moderate conductive polymer layers served as physical and chemical barriers that can remarkably suppress the shuttle effect and promote the electronic conductivity, too thick coatings may slow the ion diffusion from the electrolyte into the inner. The nonionic conductor characteristics of conductive polymers would in turn increase the interfacial contact resistance. In this regard, Jeong et al.^[^ ^108^ ^]^ incorporated a dual‐conducting poly(3,4‐ethylenedioxythiophene)‐*co*‐polyethylene glycol (PEDOT‐*co*‐PEG) to encapsulate sulfur particles (Figure 7d). Due to the dual‐conducting characteristic of PEDOT‐*co*‐PEG, the ion and electron transport of the composite can be simultaneously promoted, and consequently improve the rate capability. As expected, the sulfur cathode with only 1 wt% PEDOT‐*co*‐PEG layer delivers a reversible capacity more than 500 mAh g^−1^ at a large rate of 5C, much larger than that (only ≈100 mAh g^−1^) of pristine sulfur. Moreover, the composite cathode demonstrates an excellent cycling stability with a reversible capacity of 1002 mAh g^−1^ after 100 cycles at 0.2C due to the protection of PEDOT‐*co*‐PEG coating layer. In summary, to maximize the ratio of sulfur to polymers in the composites without sacrificing the rate capacity and cycle life, the design of core–shell structure can be further optimized with an emphasis on the thickness of polymers and the compromise of electron and ion transport. Besides, engineering traditional conductive polymers is also feasible in simultaneously improving the LiPS trapping and promoting the reaction kinetics with the low content of polymers.

#### 3.2.2 Polysulfide Trapping Polymers

Although conductive polymers exhibit effective sulfur encapsulation, sulfur may inevitably precipitate on the outer surface of the carrier in most case, which would lead to the fast capacity decay in the initial few cycles. Additional treatments such as heating or carbon disulfide washing are commonly needed to remove the inhomogeneous S on the surface.^[^ ^109^ ^]^ Therefore, designing appropriate polymers carriers with functional groups to reinforce the chemical encapsulation for sulfur and LiPS trapping could be a potential pathway to promote the electrochemical performance.^[^ ^110^, ^111^ ^]^

**Figure 8:**
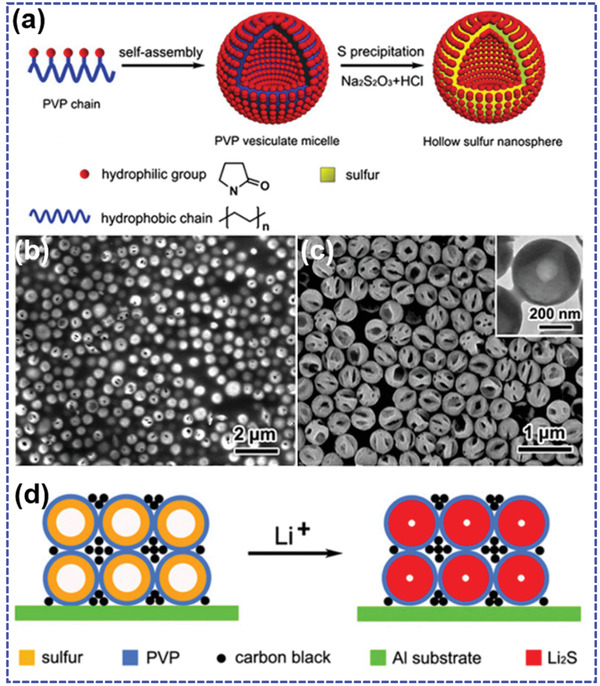
a) The fabrication process of PVP@S, scanning electron microscope (SEM) images of b) PVP‐@S and c) hollow S nanospheres after removing the PVP layers, and d) the electrode thickness evaluation of PVP@S electrode upon lithiation. Reproduced with permission.^[^ ^112^ ^]^ Copyright 2013, National Academy of Sciences.

Li et al.^[^ ^112^ ^]^ have early synthesized a polyvinylpyrrolidone (PVP)‐encapsulated hollow S nanosphere (PVP@S) through one‐step and bottom‐up method under room temperature (**Figure** **8**a). PVP not only serves as a capping agent to control the growth of sulfur particles with monodispersity, but also functions as a soft template for the formation of hollow structure. Due to the highly hydrophobic characteristic of sulfur, PVP micelle with hydrophobic alkyl portion can firmly absorb sulfur particles toward the interior of the micelle wall. PVP‐encapsulated S thus demonstrates highly monodisperse particle size with hollow interior structure (400–500 nm) due to the protection and isolation from the water (Figure 8b,c). As expected, the PVP@S cathode exhibits gradual increased capacity in the first few cycles, which can be accounted for the electrochemically active process due to the protection of PVP shell. It delivers a large capacity up to 1179 mAh g^−1^ at C/10. It is especially worth mentioning that this composite cathode demonstrates a surprising long‐term cycling stability with the capacity decay of only 0.046% per cycle during 1000 cycles at C/2 with an average Coulombic efficiency of up to 98.5%. The strong interaction between PVP and S results in the robust LiPS trapping, thereby resulting in the outstanding cycling stability. Meanwhile, the volume expansion of sulfur is spatially confined inside the hollow nanosphere due to the protection of PVP layers with intrinsic mechanical rigidity (Figure 8d). To be noticed, the electrode thickness has no change during lithiation process, which ensures the electrode integrity, avoids the loss of active sulfur, and prevents the escape of polysulfides. This work proposed an effective strategy to achieve effective polysulfide trapping by combing well‐designed nanostructure with functional polymer carriers. Since then, numerous polymers such as polydopamine,^[^ ^113^ ^]^ poly‐(sodium *p*‐styrenesulfonate),^[^ ^114^ ^]^ poly(ethylene oxide),^[^ ^115^ ^]^ polyvinylidene fluoride (PVDF),^[^ ^116^ ^]^ and polyimides^[^ ^117^ ^]^ have been successfully explored to serve as serve sulfur carriers and result in efficient LiPS trapping ability.

#### 3.2.3 Conjugated Porous Polymers (COPs)

COPs composed of functionalized building blocks have been widely applicated in electrochemical energy storage due to their porous properties.^[^ ^34^ ^]^ When served as sulfur carriers, their large specific surface area and tunable pore structure can provide sufficient space for the sulfur loading and inhibit the dissolution of LiPS through physical confinement, thus exhibiting tremendous potential in LSBs.^[^ ^118^, ^119^ ^]^ LiPS can be also chemically linked into the covalent backbone by designing building units at molecular level, resulting in securely chemical immobilization. Besides, the stable conjugated framework can bear the huge volume change during discharge/charge process.^[^ ^120^ ^]^

**Figure 9:**
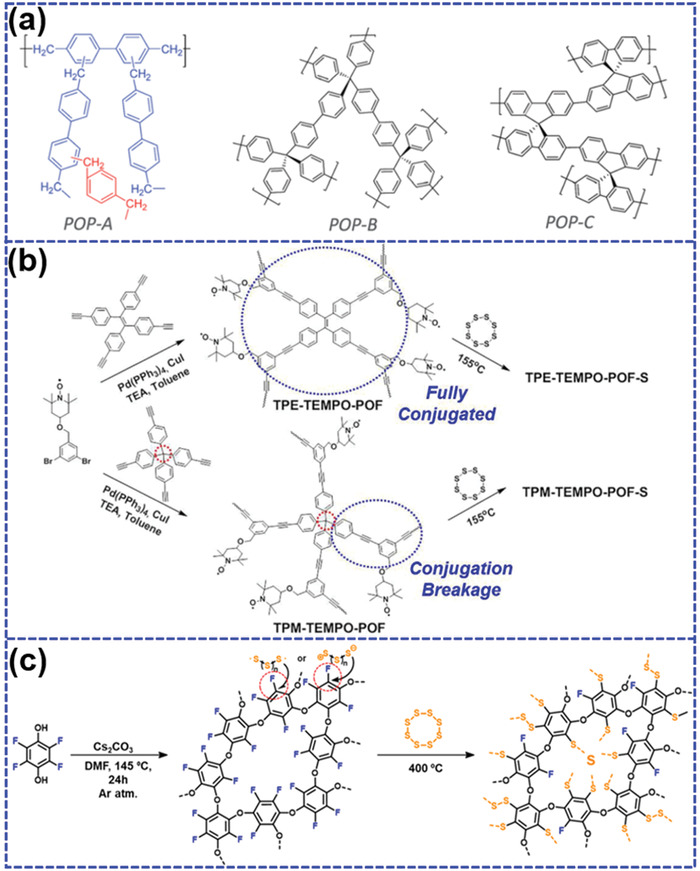
a) The molecular structures of conjugated porous polymers (POP‐A, POP‐B, and POP‐C). Reproduced with permission.^[^ ^121^ ^]^ Copyright 2014, Royal Society of Chemistry. b) The synthetic routes of TPE‐TEMPO‐POF‐S and TPM‐TEMPO‐POF‐S composites. Reproduced with permission.^[^ ^122^ ^]^ Copyright 2017, Wiley‐VCH. c) The synthetic route of fluorinated covalent organic polymer (F‐COP)/S composite. Reproduced with permission.^[^ ^123^ ^]^ Copyright 2019, American Chemical Society.

Weng et al.^[^ ^121^ ^]^ demonstrated a kind of porous organic polymer/sulfur composite (POP/S) through a facile ball milling process with a following thermal treatment (**Figure** **9**a). Three crosslinked polymers (POP‐A, POP‐B, and POP‐C) with different molecular structures all exhibit large specific areas over 1000 m^2^ g^−1^ with the pore size between 1 and 3 nm. The formed 3D network with micropores allows sufficient and homogeneous impregnation of the molten sulfur. All POP‐S cathodes demonstrate superior reversibility with the high Coulombic efficiency throughout the 100 cycles, indicating that LiPS dissolution was successfully suppressed due to the restraint of unique microporous structure. The influence of conjugation effect on the electrochemical performance for COP/sulfur composite cathode was also investigated. Zhou et al.^[^ ^122^ ^]^ synthesized two 2,2,6,6‐tetramethylpiperidine‐1‐oxyl (TEMPO)‐based porous organic frameworks with fully conjugate structure (TPE‐TEMPO‐POF) and conjugation breakage (TPM‐TEMPO‐POF) (Figure 9b). The sulfur can firmly embed into the porous channel via physical adsorption and chemical bonding through a facile melt‐diffusion process. The resulted TPE‐TEMPO‐POF‐S shows higher sulfur content of 61 wt% with the larger pore volume (0.62 cm^3^ g^−1^) than that (49 wt%) of TPM‐TEMPO‐POF‐S (0.55 cm^3^ g^−1^). Therefore, TPE‐TEMPO‐POF‐S exhibits a superior rate capability (525 mAh g^−1^ at 1C) and outstanding cycling stability (300 cycles). By contrast, TPM‐TEMPO‐POF‐S demonstrates dramatic capacity decay in only 50 cycles, which may be attributed to the blocked electron transfer due to the interrupted conjugated structure.

It has been recognized that small pores may limit the impregnation of sulfur, and then decrease the energy density although they can effectively inhibit the dissolution of LiPS. On this account, Shin et al.^[^ ^123^ ^]^ designed a fluorinated covalent organic polymer (F‐COP) as sulfur carrier through nucleophilic aromatic substitution reaction (SNAr) (Figure 9c). The F‐COP shows a predominantly microporous structure with a high specific area of 546 m^2^ g^−1^, which provides sufficient absorption sites for sulfur impregnation. Moreover, the aromatic fluorines can readily realize the covalent attachment of sulfur due to the facile F‐to‐S substitution, thereby enabling a high sulfur content over 70 wt%. The well‐defined ultramicroporous F‐COP with the major pore sizes below 2 nm exhibits strong affinity to LiPS, thereby effectively suppressing the shuttle effect. One of the highlights of this work is the simultaneous realization of high sulfur contents and effective LiPS encapsulation via steric and electrostatic hindrance. As expected, F‐COP/S composite shows a large capacity of 1287.7 mAh g^−1^ at 0.05C with a high sulfur loading up to 4.1 mg_sulfur_ cm^−2^. Moreover, it demonstrates a capacity retention up to 70.3% even after 1000 cycles at 0.5C with an average Coulombic efficiency of 99.96% throughout the cycling, suggesting an unprecedented cycling stability.

#### 3.2.4 Organic Frameworks

Compared to amorphous porous organic polymers, covalent organic frameworks (COFs) as a kind of crystalline organic framework can not only supply sufficient sites for ample sulfur infiltration and physical LiPS absorption, but also facilitate fast charge/ionic transport through the highly ordered nanopores, thereby leading to promoted redox kinetics.^[^ ^118^, ^124^ ^]^ Moreover, selecting suitable linking groups such as imines, boroxines, and triazines can strengthen the interactions between sulfur species and heterogeneous composition, thereby promoting the chemical anchoring to LiPS.

**Figure 10:**
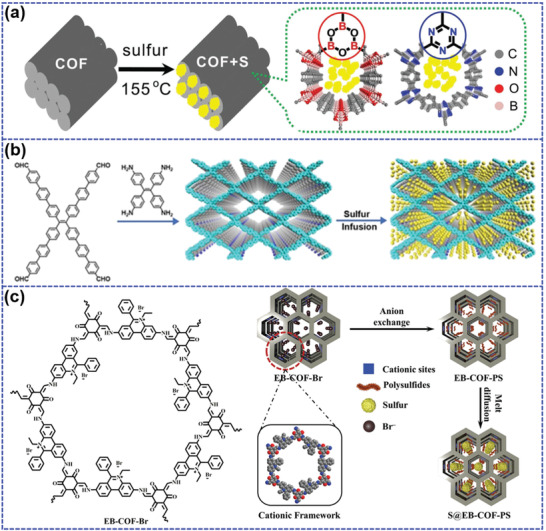
a) The synthetic route and chemical structure of N and B doped COF/S composites. Reproduced with permission.^[^ ^125^ ^]^ Copyright 2016, Wiley‐VCH. b) The preparation process of COF‐ETTA‐ETTCA‐S composite. Reproduced with permission.^[^ ^126^ ^]^ Copyright 2020, American Chemical Society. c) The molecular structure of EB‐COF‐Br and synthetic route of S@EB‐COF‐PS. Reproduced with permission.^[^ ^127^ ^]^ Copyright 2020, Wiley‐VCH.

Ghazi et al.^[^ ^125^ ^]^ synthesized a boronate ester‐based COF (B‐doped COF) and used it as sulfur carrier through simple melt‐diffusion method (**Figure** **10**a). B‐doped COF with the pore size of 1.5 nm displays uniform sulfur distribution in order nanopore. Compared to N‐doped COF,^[^ ^128^ ^]^ B‐doped COF possesses stronger adsorption ability to LiPS due to the coexistence of positively B and negatively O in the polymer matrix, which provide stronger chemical interaction between LiPS and polymer. Therefore, the shuttle effect can be remarkably inhibited by the synergistical confinement of physical adsorption and chemical encapsulation. The B‐doped COF/S shows an initial capacity up to 1628 mAh g^−1^ at 0.2C and maintains a reversible capacity of 929 mAh g^−1^ after 100 cycles. Lu et al.^[^ ^126^ ^]^ demonstrated a 2D COF with extended *π*‐conjugated units (COF‐ETTA‐ETTCA) as sulfur carrier (Figure 10b). COF‐ETTA‐ETTCA exhibits a large specific area of 490 m^2^ g^−1^ with the major pore size of 1.31 nm, which provides open nanochannels for sulfur incorporation. Herein, sulfur is only physical absorbed into the COF skeleton with the loading up to 88.4 wt% rather than chemical bonding. Nevertheless, LiPS can be effectively captured by COF matrix via the interaction between N atoms and Li‐ions.^[^ ^125^ ^]^ Meanwhile, both two conjugated sites (tetraphenylethene units with imine and phenyl rings) show strong entrapment ability to LiPS species. The small‐sized LiPS can be also stabilized within 2D interlayer with well‐fitted interlayer space (4.8 Å), further enhancing better adsorption. Additionally, the extended *π*–*π* conjugated chains can accelerate electron transport compared to nonconjugate structures, hereby ameliorating the electrochemical reaction kinetics. Benefiting from these virtues, COF‐ETTA‐ETTCA‐S composite cathode delivers a large capacity of 1617 mAh g^−1^, which is almost close to the theoretical capacity. It also demonstrates an outstanding polysulfides trapping with an extremely low capacity decay of 0.077% per cycle and high Coulombic efficiency up to 98.0% throughout 528 cycles at 0.5C.

In spite of the above breakthrough of COF‐based sulfur cathodes, their current electrochemical performances are still barely satisfactory due to the insufficient active centers for LiPS anchoring. Recently, Liu et al.^[^ ^127^ ^]^ reported a cationic COF (EB‐COF‐Br) composed of ethidium bromide (EB) and 1,3,5‐triformylphloroglucinol (Tp) as LiPS reservoir (Figure 10c). In order to remove the steric hindrance of counter ion Br^−^, EB‐COF‐Br first underwent an anion exchange process in Li_2_S_8_ solution to form EB‐COF‐PS. Then, S@EB‐COF‐PS composite with 71.7 wt% sulfur content was obtained through a melt diffusion process. Different from traditional COF, EB‐COF‐Br exhibits strong interaction with LiPS due to the incorporation of cationic quaternary ammonium sites. These cation sites can allow additional LiPS adsorption by reducing the electron density. It can also act as a bridge between electron input and LiPS output during discharge process, thus reducing the kinetic barrier. As a result, S@EB‐COF‐PS delivers a large capacity of 1136 mAh g^−1^ at 0.1C. When the rate increased to 4C, the reversible capacity can be still maintained about 686 mAh g^−1^, much larger than that (173 mAh g^−1^) of S@TpPa without cation sites, suggesting a rapid electron transfer and higher sulfur utilization. After 200 cycles at 4C, the S@EB‐COF‐PS keeps a high capacity of 468 mAh g^−1^, suggesting a superb cycling stability.

Overall, compared to other polymer carriers, the physical adsorption and chemical trapping to LiPS, high sulfur loading with homogeneous distribution and enhanced reaction kinetics are expected to be simultaneously achieved by customizing specific building units and linking groups at molecular level for COP‐ and COF‐based sulfur cathodes. The adjustable specific area and pore structure, designable functional group, tunable topological structure, and conjugation effect of porous polymers with amorphous or crystalline characteristic leave much room for the improvement of their electrochemical performances.

### 3.3 Polymer‐Based Composites Carriers for Sulfur

#### 3.3.1 Carbon/Polymer Composites

The foregoing parts have manifested that well‐designed polymer carriers can achieve effective LiPS trapping. Nevertheless, the poor rate capability caused by the electrical insulation of elemental sulfur is another issue that cannot be ignored. It is noted that carbonaceous materials such as graphene,^[^ ^129^, ^130^ ^]^ carbon nanotubes,^[^ ^131^, ^132^ ^]^ carbon black,^[^ ^133^ ^]^ and porous carbon^[^ ^134^ ^]^ are ideal carrier candidates due to their high electronic conductivity. Therefore, the incorporation of multicomponent carbon/polymer composites served as sulfur carriers is regarded as an optimized way to simultaneously promote the rate capability and cycling stability of sulfur cathodes.

**Figure 11:**
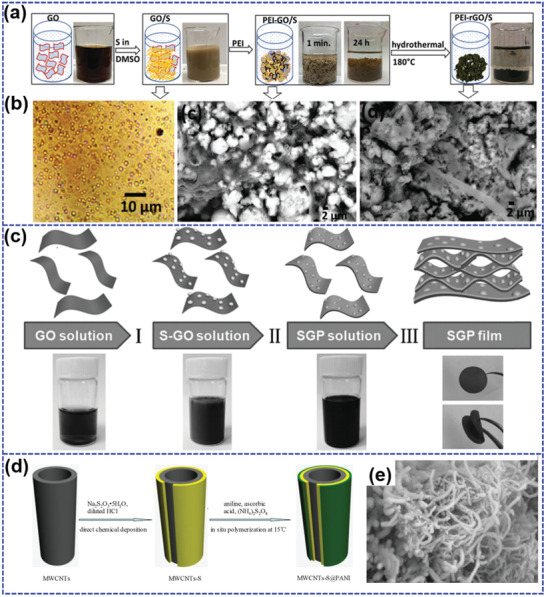
a) The synthetic route of PEI–rGO/S composite and b) the corresponding morphology. Reproduced with permission.^[^ ^135^ ^]^ Copyright 2018, Wiley‐VCH. c) The fabrication process of binder‐free nanosulfur/graphene/PEDOT:PSS (SGP) film. Reproduced with permission.^[^ ^136^ ^]^ Copyright 2017, Wiley‐VCH. d) The schematic illustration and e) SEM image of MWCNTs‐S@PANI. Reproduced with permission.^[^ ^131^ ^]^ Copyright 2015, Elsevier.

Ghosh et al.^[^ ^135^ ^]^ reported a partially reduced graphene oxide (rGO) and crosslinked polyethyleneimine (PEI–rGO) as sulfur carrier and LiPS reservoir through a modified hydrothermal method (**Figure** **11**a). Although rGO with high specific area provides sites and space for the adsorption of sulfur, the impregnated sulfur can easily nucleate and reaggregate due to the Brownian motion. The previous reports have proved that PEI can covalently interact with functionalized carbon and polar LiPS.^[^ ^137^ ^]^ Herein, the cationic PEI with rich amine groups can link negatively charged rGO to form PEI–rGO network and recapture the amorphous sulfur. Besides, PEI and sulfur can also function as spacer to prevent the restack of rGO during the hydrothermal treatment. Therefore, sulfur with the content of 75.7 wt% and areal loadings up to 5.4 mg cm^−2^ can be highly dispersed through the PEI–rGO network (Figure 11b). During the discharge/charge process, the crosslinked PEI–rGO network can buffer the huge volume change of element sulfur. Moreover, the amino groups of PEI and functionalized rGO with C—S/C═S groups can chemically interact with polar LiPS, resulting a significantly inhibited shuttle effect. The PEI–rGO/S cathode hence shows an outstanding cycling stability with negligible capacity decay after 250 cycles at 0.15C. Even after 810 cycles at 0.75C, there is only 0.028% capacity decay rate per cycle.

Although the carbon/polymer carriers can lead to promoted cycle life and fast kinetic, the presence of electrochemical inactive components and the formed void space existing in the multicomponent carriers would decrease the volumetric capacity, which plays a key role for the practical application of LSBs.^[^ ^48^ ^]^ On this account, Xiao et al.^[^ ^136^ ^]^ fabricated a flexible and free‐standing sulfur cathode (SGP) through a facile vacuum filtration method (Figure 11c). Nano‐S was first in situ homogenously deposited on the surface of graphene oxide (GO) sheets due to their functional groups and large surface area. Then, PEDOT:PSS were adsorbed tightly on the GO surface due to the *π*–*π* interaction between PEDOT and 2D graphene sheets. The resulted SGP film with sandwich structure exhibits a high electronic conductivity of 36.5 S cm^−1^, which almost keeps unchanged (35.9 S cm^−1^) even after being bent for 100 times, suggesting a superior mechanical flexibility. As a result, the SGP film shows a large initial capacity of 1584 mAh g^−1^ at 0.1C and maintains a capacity of 701 mAh g^−1^ at a large rate 4C, indicating a superior rate capability. It also displays an excellent cycle life with the capacity retention of 80% after 500 cycles at 1C. The absorption tests reveal that PEDOT:PSS and GO can synergistically LiPS through strong chemical interaction. Due to the absence of inactive binder and less void space in the electrode, the compact sandwich structure endows SGP high volumetric sulfur loading (1 g cm^−3^), thereby achieving a large volumetric capacity (1432 Ah L^−1^).

Similar to graphene, carbon nanotubes with high electronic conductivity and large specific surface area have been also employed to fabricate sulfur/carbon/polymer composite cathodes.^[^ ^110^, ^138^ ^]^ Li et al.^[^ ^131^ ^]^ synthesized a PANI coated sulfur grown on multiwalled carbon nanotubes (MWCNTs‐S@PANI) through chemical precipitation with a following in situ polymerization process (Figure 11d). The 3D network of carbon nanotubes is well preserved after the deposition of sulfur and coating of PANI (Figure 11e). MWCNTs‐S@PANI with 67.8 wt% sulfur content displays a reversible capacity of 545.5 mAh g^−1^ after 205 cycles at 0.2C, much larger than that (353.4 mAh g^−1^) of MWCNTs‐S without PANI coating layers. The increased capacity and boosted stability are ascribed to the collaboratively promotion of PANI and MWCNTs, which not only greatly boosts the electronic conductivity, but also effectively inhibits the dissolution of LiPS.

#### 3.3.2 Metallic Compound/Polymer Composites

Single unfunctionalized polymers as sulfur carriers still suffer from the limited trapping ability to due to the weak polar–polar interaction. Furthermore, the poor wettability and low contact area with electrolyte of most polymers also lead to inadequate LiPS immobilization. In comparison, metallic compounds with strong polarity such as metal oxides,^[^ ^139^, ^140^ ^]^ metal sulfides,^[^ ^141^ ^]^ Prussian blue analogues,^[^ ^142^ ^]^ and metal–organic frameworks^[^ ^143^ ^]^ have been regarded as ideal sulfur carriers for their robust affinity to LiPS via strong Lewis acid–base interaction.

**Figure 12:**
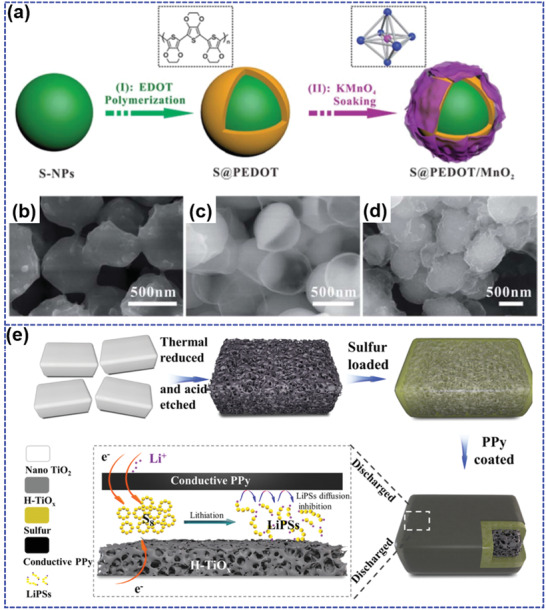
a) The synthesis schematic of S@PEDOT/MnO_2_ composite, and SEM images of b) sulfur nanoparticles (S‐NPs), c) S@PEDOT, and d) S@PEDOT/MnO_2_. Reproduced with permission.^[^ ^144^ ^]^ Copyright 2016, Royal Society of Chemistry. e) The synthesis schematic of H‐TiOx@S/PPy composite. Reproduced with permission.^[^ ^145^ ^]^ Copyright 2019, American Chemical Society.

Yan et al.^[^ ^144^ ^]^ fabricated MnO_2_ nanosheet functionalized sulfur@PEDOT core–shell nanoparticles (S@PEDOT/MnO_2_) as LSBs cathode via two‐steps coating processes (**Figure** **12**a–d). The inner PEDOT layer on the surface of as‐prepared sulfur nanoparticles can not only promote the electrical conductivity, but also function as a protective layer to retard the migration of LiPS. The outer MnO_2_ layer can further entrap partially escaped LiPS from the inside through chemical interaction between the hydrophilic Mn–O/hydroxyl groups and LiPS.^[^ ^146^ ^]^ In addition, the amorphous MnO_2_ nanosheets can also enhance the wettability with the electrolyte and effectively adsorb Li‐ions due to the large active surface area, resulting in an improved reaction kinetics. Based on the above superiorities, S@PEDOT/MnO_2_ with a high sulfur content of 87 wt% delivers a large capacity of 1164 mAh g^−1^ at 0.2C and maintains 631 mAh g^−1^ at a high rate of 3C, much larger than that (297 mAh g^−1^ at 3C) of S@PEDOT without MnO_2_ layer, suggesting a superior rate capability. In particular, S@PEDOT/MnO_2_ displays an enhanced cycling stability with the reversible capacity of 827 mAh g^−1^ even after 200 cycles at 0.2C compared to that (551 mAh g^−1^) of S@PEDOT. Chen et al.^[^ ^145^ ^]^ also proposed a multifunctional sulfur carrier by combing defect‐abundant H‐TiO*_x_* (*x* = 1, 2) and conductive PPy (Figure 12e). H‐TiO*_x_* matrix was initially served as sulfur carrier (H‐TiO*_x_* @S), then PPy layers was coated on the surface of H‐TiO*_x_* @S through an in situ polymerization process. The resulted H‐TiO*_x_* @S@PPy exhibits a sulfur content of 66.98 wt%. The electrochemical performance tests show that H‐TiO*_x_* @S@PPy delivers large capacities of 1130 and 726 mAh g^−1^ at 0.1C and 1C, respectively. Even after a long‐term 1000 cycles, the capacity can still maintain about 411.7 mAh g^−1^ at 1C with an extremely low capacity decay rate of 0.0406% per cycle, behaving an excellent cycling stability. The superior performance mainly profits from the synergistical boost of H‐TiO*_x_* and PPy. The H‐TiO*_x_* can chemically anchor LiPS through the formation of Ti—S bond via Lewis acid–base interaction. The outer PPy layer can also hinder LiPS dissolution trough strong polar–polar (Li—N bond) interaction and physical absorption. Besides, the abundant defects of H‐TiO*_x_* and high conductivity of PPy can enhance the electron transfer, thereby greatly promoting the reaction kinetic and sulfur utilization.

Consequently, the above work confirms that the combination of polar metallic compounds and well‐designed polymers as multifunctional sulfur carriers can simultaneously address the insulation of sulfur and discharged products, suppress the volume change and shuttle effect. Future work should be continuously focused on the functional design of polymers, the modification of metallic compounds, and the rational construction of multicomponent nanostructure to further reinforce LiPS anchor, enhance the kinetics, and promote the sulfur content with high surface area loading.

## 4 Polymer Binders in Cathodes

Apart from the active materials, the binder also plays an irreplaceable role in optimizing the electrochemical performance of LSBs, although it generally only occupies a small portion (<10 wt%) in the electrode components.^[^ ^147^, ^148^ ^]^ The binder can adhere active materials and conductive carbon to ensure continuous electrical contact. Meanwhile, it guarantees robust adhesion of all the components on the current collector, thereby holding the electrodes stability. As for LSBs, well‐designed binders can also improve the dispersion state inside the electrodes, consequently resulting in enhanced inhibition of LiPS dissolution. Therefore, the binders for LSBs should not only have excellent adhesion, structural/mechanical, and electrochemical stability, but also immobilize the LiPS to suppress the shuttle effect.^[^ ^149^ ^]^

### 4.1 Fluoro‐Polymers

Currently, PVDF binder has been successfully commercialized in traditional LIBs due to its strong adhesion property and superior chemical stability in the organic electrolyte.^[^ ^154^ ^]^ Unfortunately, PVDF binder generally exhibits dissatisfied electrochemical performance when used in LSBs. First, the weak adhesion of PVDF based on van der Waals forces is not enough to withstand huge volume expansion of sulfur cathode and maintain the electrode integrity during discharge/charge process.^[^ ^149^, ^155^ ^]^ Then, the swellability of PVDF in ether‐based electrolytes would restrict the penetration of electrolyte into the electrodes, leading to rapid capacity degradation.^[^ ^156^ ^]^ Besides, the electron‐withdrawing —CF groups of PVDF have insufficient binding energy to LiPS. Therefore, the exploration of novel binders with high adhesion property, excellent mechanical/chemical stability and strong chemical interaction with LiPS has been a challenge for the practical application of LSBs.

**Figure 13:**
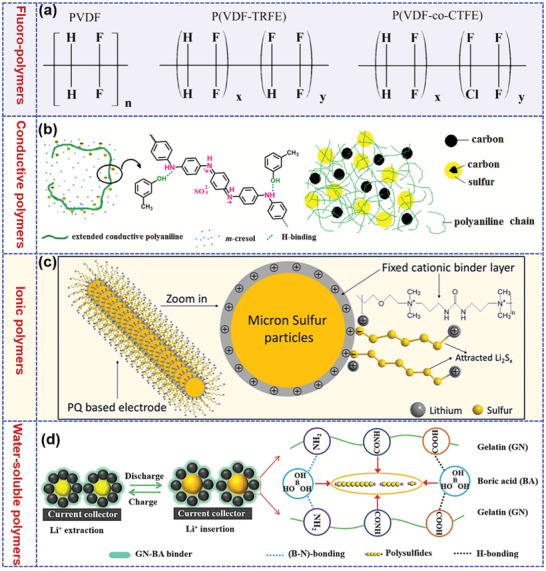
Polymer binders for LSBs: a) The molecular structures of PVDF, P(VDF‐TRFE), and P(VDF‐*co*‐CTFE) binders. Reproduced with permission.^[^ ^150^ ^]^ Copyright 2016, Elsevier. b) The molecular structure and schematic of conducting polyaniline binder. Reproduced with permission.^[^ ^151^ ^]^ Copyright 2017, Elsevier. c) The chemical structure and polysulfides confinement of cationic poly[bis(2‐chloroethyl) ether‐alt‐1,3‐bis[3‐(dimethylamino) propyl]urea] quaternized (PQ) binder. Reproduced with permission.^[^ ^152^ ^]^ Copyright 2017, American Chemical Society. d) The schematic diagram of water‐soluble GN–BA crosslinked binder. Reproduced with permission.^[^ ^153^ ^]^ Copyright 2021, Wiley‐VCH.

In order to improve the adhesive strength and affinity of the binder to LiPS, Wang et al.^[^ ^150^ ^]^ employed two modified fluoro‐polymer binders [P(VDF‐TRFE) and (P(VDF‐*co*‐CTFE)] for mesoporous carbon/sulfur cathodes (**Figure** **13**a). Density functional theory (DFT) calculations clearly reveal that P(VDF‐TRFE) with more fluorine atoms exhibits higher binding energy to LiPS than PVDF with only two fluorine atoms, suggesting a stronger affinity. As for P(VDF‐*co*‐CTFE), the replacement of hydrogen in the trifluorethylene by chloride atom results in an enhancement of molecular polarity. The adhesive property tests confirm that both P(VDF‐TRFE) and P(VDF‐*co*‐CTFE) show better adhesion strength than PVDF. As a result, S@P(VDF‐TRFE) and S@P(VDF‐*co*‐CTFE) cathodes deliver larger capacities and better rate capabilities than homopolymer PVDF binder. It is noted that P(VDF‐TRFE) demonstrates a much‐promoted cycling stability with a stable capacity of 801 mAh g^−1^ after 100 cycles at 0.2C, far better than that (450 mAh g^−1^) of the S@PVDF cathode. Additionally, the P(VDF‐TRFE) can easily fill the pores between conducive carbon and active materials, resulting in more uniform distribution of binder and sufficient accessibility of electrolyte, which has been proved in the previous report.^[^ ^156^ ^]^

### 4.2 Conductive Polymers

Traditional fluoro‐polymer binders are electronic insulators, which is not beneficial to improve the rate capability of sulfur cathodes. In comparison, conducting polymers with unique adhesive ability based on van der Waals and electrostatic interactions have been explored as alternatives to fluoro‐based binders.^[^ ^157^ ^]^ As discussed in the aforementioned parts, conductive polymers with heteroatoms can realize the chemical bonding to LiPS, which can promote the cycling stability. However, most conductive polymers appear brittleness due to the rigid conjugated skeleton, which is hardly to buffer the serious volume expansion of sulfur, thereby generally resulting in the loss of active materials.^[^ ^158^ ^]^

To address the rigidity of conductive polymer chains, Gao et al.^[^ ^151^ ^]^ synthesized a sulfuric acid doped PANI binder with cobweb‐like structure to resist the expansion of sulfur (Figure 13b). The employed *m*‐cresol can dissolve PANI to form extended chain structure through the formation of H‐bonding. The crosslinked structure can expand the contact area between active sulfur and binder, and provide sufficient space for the swelling of sulfur during discharge/charge process. Besides, acid‐doping incorporates polar groups like amine salt into the polymer backbone, which is expected to strengthen the LiPS trapping ability. The formed cobweb‐like structure can adequately twin sulfur particles together, in which leads to an extremely low portion (2 wt%) of binder in the electrode. The reduction of binder content in turn would increases the proportion of active substances in the electrode components, leading to the promoted energy density. Therefore, PANI‐based cathode exhibits the significantly improved capacity, rate capability, and cycling stability compared to traditional acrylonitrile copolymer (LA132), polytetrafluoroethylene (PTFE) and PVDF binders. Similar to PANI, PEDOT:PSS with high electrical conductivity (4600 S cm^−1^) and strong film‐forming property has been also regarded to a possible binder for sulfur cathodes.^[^ ^159^ ^]^ Yan et al.^[^ ^160^ ^]^ synthesized a crosslinked PEDOT:PSS–Mg^2+^ with a 3D network by using Mg^2+^ as crosslinker. The crosslinked network formed by the electrostatic interaction between Mg^2+^ and neighboring PSS chains notably increases the adhesion ability to the current collector. The dissolution of LiPS can be synergistically inhibited by the physical confinement of 3D network and strong chemical trapping. It is noted that the incorporation of Mg^2+^ has negligible influence on the electronic conductivity of PEDOT:PSS. Even at a large rate of 2C, the PEDOT:PSS–Mg^2+^‐based cathode maintains a stable capacity of 576 mAh g^−1^, much larger than that (410 mAh g^−1^) of the corresponding one with PVDF, indicating better rate capability. It also shows an improved capacity retention of 74% after 250 cycles at 0.5C compared to that (51%) of the cathode with PVDF binder.

### 4.3 Ionic Polymers

Exploring bifunctional polymer binders through molecular engineering plays an essential role on intensifying the adhesiveness and trapping ability to LiPS. It has been recognized that polymer carriers with rich polar groups can realize better LiPS immobilization. Therefore, the incorporation of functional groups such as imine, ketone, ester, amide, and ether in binders can be expected to enhance the binding energy to LiPS.^[^ ^161^, ^162^ ^]^ Seh et al.^[^ ^163^ ^]^ had earlier proved that polar ammonium cations have higher binding energy to LiPS than most reported functional groups. In this regard, Ling et al.^[^ ^152^ ^]^ used a cationic poly[bis(2‐chloroethyl) ether‐alt‐1,3‐bis[3‐(dimethylamino) propyl]urea] quaternized (PQ) as binder for micrometer sulfur cathodes (Figure 13c). The rich quaternary ammonium cations (R_4_N^+^) with positively charge in the PQ backbone has strong affinity to negative charged LiPS via electrostatic force. PQ‐based cathode with 60 wt% sulfur content exhibits a high sulfur utilization of 70% for the initial cycle. And a large areal capacity of 9.0 mAh cm^−2^ can be achieved at a large surface loading of 7.5 mg cm^−2^. It is noted that PQ‐based cathode demonstrates two well‐defined discharge plateaus, corresponding to typical LSBs mechanism and suggesting a well‐controlled shuttle effect. In comparison, PVDF‐based cathode with same sulfur loading only displays the sulfur utilization of 13%, which fast decreases to 5% after 30 cycles. And, the disappearance of the second discharge plateau at the lower potential can be accounted for the failure trapping of long‐chain LiPS.

### 4.4 Water‐Soluble Polymers

Commercial binders represented by fluoro‐based polymers can only be dissolved in organic solvents such as *N*‐methylpyrrolidone (NMP). The employment of these organic solvents during the fabrication process of the electrode may result in serious environmental pollution and high cost due to their toxic, volatile, flammable properties. In recent years, various water‐soluble binders with hydrophilicity such as polyvinyl alcohol (PVA),^[^ ^164^ ^]^ carboxymethyl cellulose–butadiene styrene rubber (CMC–SBR),^[^ ^165^ ^]^ poly(ethylene oxide) (PEO),^[^ ^166^ ^]^ LA132,^[^ ^167^ ^]^ poly(acrylic acid) (PAA),^[^ ^168^ ^]^ carboxymethyl cellulose (CMC),^[^ ^169^ ^]^ chitosan,^[^ ^170^ ^]^ hyperbranched poly(amidoamine),^[^ ^171^ ^]^ and guar gum (GG)^[^ ^172^ ^]^ as alternatives to organic solvent soluble binders have received more attention.^[^ ^173^ ^]^ The electrode fabrication process only needs water as the solvent, which greatly reduce the cost and avoids the possible environmental pollution. Besides, these water‐soluble binders commonly exhibit strong chemical adsorption ability to LiPS due to the abundant polar groups in the polymer backbone.

Sun et al.^[^ ^153^ ^]^ recently synthesized a novel water‐soluble binder (GN–BA) through the crosslinking of gelatin (GN) and boric acid (BA) (Figure 13d). GN as a well‐known natural polymer contains rich hydrophilic group like —CONH—, —NH_2_, and —COOH, which exhibits good solubility in water. These polar groups can also effectively trap LiPS through the coordination effect. The experimental and DFT calculation investigations reveal that the chemical adsorption to LiPS through forming chemical bonds (C—O—Li, C—N—Li, and B—O—Li) plays a major role in preventing the shuttle effect. The network crosslinked by B‐OH groups has excellent structural stability due to the strong intermolecular force, which is beneficial to buffer the volume expansion of sulfur and preserve the electrode integration during cycles. GN–BA binder also exhibits stronger binding strength than PVDF binder. When served as the binder for commercial sulfur powders, S@GN–BA cathode shows better rate capability with much higher capacity of 580 mAh g^−1^ than that of S@PVDF (180 mAh g^−1^), indicating a higher sulfur utilization. Even at a large sulfur loading of 5.0 mg cm^−2^, the areal specific capacity can still reach up to 5.7 mAh cm^−2^. This work proposes an economical and environmentally friendly strategy to construct multifunctional binders for LSBs.

## 5 Polymer Interphases between the Cathode and Separator

Although elaborated carriers can confine sulfur and LiPS in a limited space via physical absorption and chemical interaction, the host materials may occur gradually structural collapse particularly for rigid inorganic carries due to the accumulation of LiPS, which is especially serious under high sulfur loading. The separators with microporous structure generally only function as an electronic insulator and lithium ions conductor, which may also allow the transportation of LiPS. Therefore, inserting an interphase between the cathode and the separator as a barrier layer becomes another approach to prevent the dissolution and migration of LiPS from the cathode to the anode.^[^ ^174^, ^175^ ^]^

**Figure 14:**
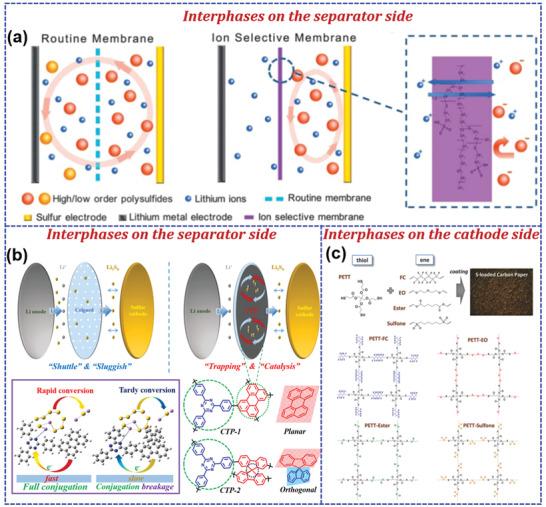
Polymer interphases between the cathode and the separator: a) The schematics of LSB configurations with routine and Nafion coated Celgard 2400 separator. Reproduced with permission.^[^ ^176^ ^]^ Copyright 2014, Royal Society of Chemistry. b) The schematics of LSB configurations with routine and CTP coated Celgard 2500 separators. Reproduced with permission.^[^ ^177^ ^]^ Copyright 2019, Elsevier. c) The schematic representation of polymer‐coated sulfur/carbon paper electrodes and the molecular structures of PETT‐FC, PETT‐EO, PETT‐ester, and PETT‐sulfone. Reproduced with permission.^[^ ^178^ ^]^ Copyright 2015, Royal Society of Chemistry.

Huang et al.^[^ ^176^ ^]^ introduced a polymer interphase between the cathode and separator by directly coating Nafion on the surface of commercial Celgard 2400 separator (**Figure** **14**a). Nafion is a well‐known tetrafluoroethylene‐based polymer with ion selectivity, which contains sulfonate (SO_3_^−^)‐terminated perfluorovinyl ether groups.^[^ ^179^ ^]^ Compared to routine separator, the Nafion decorated separator served as anion shield can prevent LiPS crossing through the channel due to the Coulombic interactions between SO_3_^−^ groups and LiPS anions. Consequently, the shuttle effect is effectively inhibited and the unfavorable side effects between the anions and lithium metal anode are also avoided. When used in LSBs, the assembled battery with CNT–sulfur cathode exhibits a significantly promoted capacity retention (>60%) with a decay rate of only 0.08% per cycle along 500 cycles at 1C, much superior to the batteries with routine separator (34.4% capacity retention and the decay rate of 0.30%). Besides, the possible polarization caused by the introduction of coating layers on the separator can be eliminated by controlling the thickness of Nafion layer. This work highlights the importance of interlayer on blocking the shuttle effect and proposed a simple method to build LiPS shield between the sulfur cathode and separator.

In order to strengthen the chemical LiPS trapping ability of the interlayers, Xu et al.^[^ ^177^ ^]^ recently built a novel polymer interlayer by using two triazine‐based frameworks (CTP‐1 and CTP‐2) to decorate commercial Celgard 2500 (polypropylene, PP) separator (Figure 14b). Herein, the CTP layer with porous skeleton not only builds a reinforced physical shield on the surface of PP separator, but also serves as additional LiPS immobilizer. CTP‐1 and CTP‐2 coated separators exhibit enhanced electrolyte wettability compared to the routine separator, which can promote the redox kinetic. It has been reported that the Lewis acid–base interaction between negative N atoms and Li‐ions can effectively trap LiPS.^[^ ^180^ ^]^ Therefore, the CTP‐1 with higher pyridinic N content possesses stronger LiPS affinity than CTP‐2. Meanwhile, CTP‐1 with fully conjugated pyrene backbone demonstrates faster electron transfer than CTP‐2 with interrupted conjugation structure. As a result, CTP‐1 modified separator presents more effective LiPS immobilization and faster kinetics, resulting in better rate capability and cycle life than CTP‐2. When assembled with S/CNT cathode, CTP‐1‐based battery delivers an outstanding rate capability (637 mAh g^−1^ at 5.0C) and a superior cycling stability with only 0.048% capacity decay rate along 800 cycles at 1.0C.

In addition to construct polymer interphase on the separator side, forming a layer directly on the cathode surface is also a feasible approach.^[^ ^181^ ^]^ Park et al.^[^ ^178^ ^]^ designed a series of highly crosslinked polymer coating layers through a facile photopolymerize process on the surface of as‐prepared S‐loaded carbon paper (Figure 14c). The elaborated polymer layers with different electron‐donating functional groups such as 1,6‐divinylperfluorohexane (FC), di(ethylene glycol) divinyl ether (EO), divinyl adipate (ester), and 1,6‐bis(vinylsulfone)hexane (sulfone) are expected to effectively bind LiPS. However, the PETT‐FC coated cathode exhibits fast capacity decay due to its highest degree of swelling. PETT‐sulfone also delivers a low capacity due to the rigidity of polymer chains, which is dimensionally unfavorable for the formation of Li_2_S. In comparison, ester group exhibits strengthened binding force and more binding sites to LiPS due to its stronger electron donating property. PETT‐ester coated sulfur cathode thus demonstrates better cycling stability without the initial capacity loss than PEIT‐EO interlayers. More importantly, PETT‐ester with high crosslinking density show low swelling degree in the electrolyte, which can further reduce the diffusional loss of LiPSs. This work confirms that the directly coating process on the cathode without involving sophisticated nanostructures provides a simple strategy to build high‐performance sulfur cathodes for LSBs.

## 6 Polymers in Separators and Electrolytes

### 6.1 Polymer Separators

Porous separators as an indispensable component in LSBs play a critical role on isolating the cathode from the anode and avoiding the short circuit.^[^ ^36^ ^]^ As separator is the only way for soluble LiPS to enter the lithium anode, reasonable design and modification of the separators is an effective approach to suppress the shuttle effect and improve the overall performance of LSBs. The currently used separators are mainly porous polyolefin membranes such as polyethylene (PE) or PP, which have been commercialized in traditional LIBs due to their high ionic conductivity, strong chemical/mechanical stability, and low cost. However, LiPS can easily migrate through traditional polyolefin separators due to their micrometer scale pore channels, giving rise to unscrupulous shuttle effect. Therefore, apart from being able to conduct Li‐ions and isolate electrons, ideal separators for LSBs should also block LiPS and limit the dissolved species to the cathode area as well.

**Figure 15:**
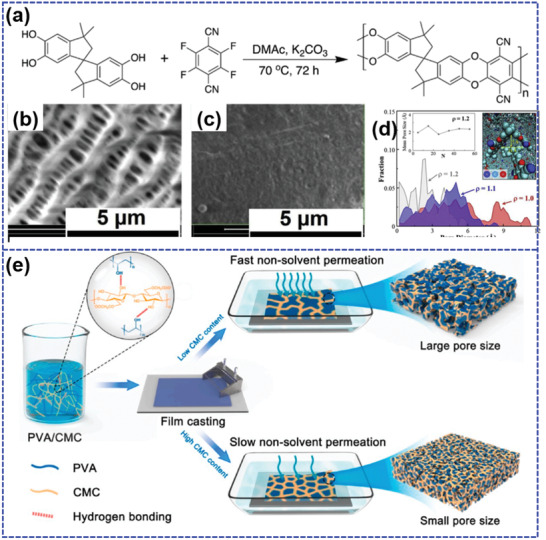
a) The synthetic route of PIN separator, SEM images of b) Celgard separator and c) PIN separator, and d) the pore size distributions of PIN separator from molecular dynamics (MD) simulations. Reproduced with permission.^[^ ^182^ ^]^ Copyright 2018, Elsevier. e) The synthetic schematic of PVA/CMC composite separators. Reproduced with permission.^[^ ^183^ ^]^ Copyright 2021, Elsevier.

From the view of size‐effect, reducing the pore size of the separator provides a feasible path to restrict the transport of LiPS.^[^ ^184^ ^]^ Yu et al.^[^ ^182^ ^]^ designed a novel polymer with intrinsic nanoporosity (PIN) with unique ion selectivity and used it as the separator to inhibit the diffusion of LiPS (**Figure** **15**a). The PIN membrane was synthesized via the polymerization of tetrahydroxy‐3,3,30,30‐tetramethyl‐1,10‐spirobisindane and tetrafluoroterephthalonitrile with a following casting process. Compared to the micrometer scale of commercial Celgard separator, the prepared PIN exhibits a uniform pore dimension with the pore size <1.0 nm (Figure 15b–d). The intrinsic nanoporosity with remarkably reduced size can allow Li‐ions transport, but prevent the diffusion of LiPS. The diffusion tests confirmed that PIN demonstrates an outstanding inhibition for the migration of LiPS species compared to Celgard separator even after a long test for ten days. As expected, when used as the separator for carbon nanofiber paper loaded sulfur cathodes, PIN‐based batteries show similar initial capacities to Celgard‐based batteries, suggesting that PIN separator has a high Li‐ion conductivity. However, Celgard‐based battery demonstrates a fast capacity decay with only 49.1% capacity retention after 100 cycles at C/5, which becomes merely 23.3% when prolonged to 200 cycles. In contrast, PIN‐based battery exhibits a significantly improved capacity retention up to 75.6% even after 200 cycles. The results successfully verify the feasibility to suppress the shuttle effect through designing novel separators with intrinsic nanoporosity.

Inspired from the influence of interphases between the cathode and separator, designing new type of separators with polar functional group such as —SO_3_^2−^ and —COO— may be another effective strategy to simultaneously realize Li‐ions conduction and inhibited pass of LiPS. Recently, a porous PVA and sodium CMC composite separator (PVA/CMC) was fabricated through a nonsolvent induced phase separation process (Figure 15e).^[^ ^183^ ^]^ The PVA/CMC composite exhibits 3D nanochannels, and the pore size decreases with the increase of CMC content. The PVA/CMC separator also exhibits higher porosity and faster Li‐ion diffusion than PP separator due to its 3D nanochannels. As a result, the PVA/CMC assembled battery delivers a larger capacity 1392 mAh g^−1^ at 0.1C, which maintains about 523.6 mAh g^−1^ at a large rate of 5C, implying much batter rate capability than that of PP separator. To be noticed, due to the presence of abundant carboxylate groups in CMC with negative charge, the PVA/CMC separator demonstrates efficiently suppressed LiPS migration. After 500 cycles at 1C, the reversible capacity of PVA/CMC assembled battery can still achieve up to 520.9 mAh g^−1^ with only 0.045% capacity decay rate. Meanwhile, the Coulombic efficiency can always keep above 99.1% along the cycles. The above work manifest that reasonable design of multifunctional separators from the perspective of size effect and molecular functionalization can be expected to facilitate Li‐ions transfer and suppress the shuttle effect.

### 6.2 Polymer Electrolytes

Traditional liquid organic electrolytes have been successfully utilized in commercial LIBs due to their high ionic conductivity and good interface contact. They also demonstrate similar properties in the current LSBs. However, the low Li‐ions migration number, easy leakage, volatile, and flammable characteristics of liquid organic electrolytes would result in possible safety hazards in practical application. Moreover, the dissolution of LiPS intermediates in liquid electrolytes should be mainly responsible for the shuttle effect.^[^ ^185^ ^]^ In contrast, polymer electrolytes are regarded as the most likely alternative to liquid electrolyte due to their enhanced safety without leakage, superior mechanical properties, good processability, and low cost. Meanwhile, polymer electrolytes can eliminate the possible short‐circuit caused by the growth of lithium dendrites because of the compatibility between the electrolyte and lithium anode. More importantly, polymer electrolytes with a small amount of liquid solvents can favorably reduce the dissolution of LiPS and essentially eliminate the shuttle effect.^[^ ^186^ ^]^ Besides, polymer electrolytes can function as a reservoir to immobilize LiPS within the polymer backbone.

Polymer electrolytes can be divided into two categories including all solid polymer electrolytes (SPEs) and gel polymer electrolytes (GPEs). SPEs generally consist of the polymer matrix and lithium salt, which completely avoids the use of liquid solvents, thereby thoroughly eliminating the dissolution of LiPS.^[^ ^187^, ^188^ ^]^ The Li‐ion conduction ability is provided by the dissolved lithium salt in polymer matrices through the coordination between functional groups and Li‐ions. The ion transport is realized via the mobility of polymer chains located in the amorphous region above the glass transition temperature (*T*_g_). In view of this, the choice of polymer matrix with low crystallinity plays an essential role in promoting the ionic conductivity of SPEs. Among various reported SPEs including PEO,^[^ ^189^ ^]^ PAN,^[^ ^190^ ^]^ and PVDF,^[^ ^191^ ^]^ PEO matrix with abundant polar groups such as —O— and —C═O shows high solvability to lithium salts, which has received the most extensive attention. Unfortunately, the early reported PEO‐based SPEs exhibited low ionic conductivity (10^−7^ S cm^−1^) under room temperature due to the high crystallinity and low‐portion amorphous domains in the polymer chains.^[^ ^192^ ^]^ Latest reports have confirmed that adding plasticizer into the PEO matrix is an effective way to decrease its crystallinity and *T*_g_.^[^ ^193^ ^]^ The most commonly strategy is adding inorganic fillers such as inorganic oxides into PEO matrix,^[^ ^194^ ^]^ which can increase the amorphous domains by inhibiting the reorganization of PEO chains. Besides, reducing the crystallinity of the PEO matrix through molecular engineering such as designing block or hyperbranched PEO‐based polymers, introducing other repeating units into the PEO chain by means of copolymerization can also enhance the ionic conductivity of PEO‐based SPEs.^[^ ^195^ ^]^

GPEs is formed by introducing a small amount of liquid electrolyte into polymer matrices, which combines the high safety of SPEs and high ionic conductivity of liquid electrolyte.^[^ ^186^, ^196^ ^]^ Therefore, most GPEs exhibit similar ionic conductivity (>10^−4^ S cm^−1^) to liquid electrolyte at room temperature, much larger than that of SPEs. The polymer matrix endows GPEs superior thermal, chemical, and mechanical stability, which can suppress the formation of lithium dendrite. The incorporation of liquid components can promote the interfacial wetting ability between the electrodes and electrolyte and accelerate ion conduction, thus facilitating fast electrochemical reaction. The current reported GPEs mainly employ PEO,^[^ ^197^ ^]^ PAN,^[^ ^198^ ^]^ PVDF,^[^ ^199^ ^]^ and poly(vinylidene fluoridehexafluoro propylene) (PVDF‐HFP)^[^ ^200^ ^]^ as polymer matrices. Among them, vinylidene fluoride (VDF)‐based polymers have been proved to be the most promising polymer matrix due to their outstanding mechanical and chemical stability. The strong polar covalent —C—F bonds endow them high dielectric constant, which is beneficial to the ionization of lithium salt and contributes to high carrier concentration.^[^ ^201^ ^]^ However, the previously reports indicated that PVDF‐based GPEs commonly exhibited poor trapping stability to LiPS intermediates due to their inevitable dissolution in liquid phase, resulting in unsatisfactory cycle performance.^[^ ^202^ ^]^ Meanwhile, the unstable interface between PVDF‐based GPEs and lithium metal anode also would result in poor cycling stability.^[^ ^186^ ^]^ Besides, PVDF with high crystallinity generally possess poor chain mobility, consequently leading to low Li‐ions transportation number. By contrast, PVDF‐HFP with the introduction of hexafluoropropylene (HFP) units in polymer skeleton remarkably decreases the crystallinity of PVDF, thereby greatly improves the ionic conductivity.^[^ ^203^ ^]^ Nevertheless, the polysulfide absorption ability of PVDF‐HFP‐based GPEs still needs to be strengthened when used in LSBs, which can be realized by introducing polar segments into PVDF‐HFP backbone through blending methods or copolymerizing with polar monomers.

Nevertheless, in order to promote the practical application of polymer electrolytes, the ionic conductivity as a key factor still needs to be improved in the future. Besides, most polymer electrolytes possess the limited electronic conductivity, which would result in insufficient conversation reactions from LiPS to S timely and completely during the cycling process. In addition, when the adsorption sites of polymer electrolytes are excessive, the inactive parts reduce the energy density of LSBs. Hence, figuring out the optimal amount adsorbed LiPS is of significance to design functional polymer electrolytes.

## 7 Conclusions and Perspectives

LSBs have been recognized as one of the most promising next‐generation secondary batteries. However, the successful commercialization of LSBs has been subjected to the poor cycling stability caused by the dissolution of polysulfides, poor electronic conductivity, and huge volume change of sulfur cathodes. In spite of the significant breakthroughs achieved in the past few decades, the electrochemical performance of LSBs still has ample room for improvement from all aspects of battery components. In particular, polymers demonstrate promising application prospects in LSBs due to their structural designability and functional diversity. In this review, we elaborated the working principle and challenges of LSBs, and comprehensively overviewed the latest progress in the applications of polymers in LSBs in terms of sulfur cathodes, binders, interphases between the cathode and separator, separators, and electrolytes. The polymer‐based cathodes including in organosulfur polymers and polymer‐based carriers for sulfur are particularly discussed from the viewpoint of molecular engineering.

**Figure 16:**
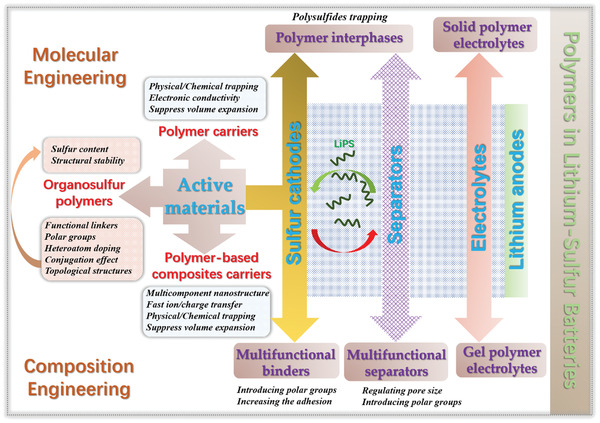
Summary and perspectives of polymers in improving electrochemical performance of LSBs.

Considering the aforementioned challenges, further exploration toward practical applications of LSBs should be focused on the following several aspects as demonstrated in **Figure** **16**. First, the sulfur cathode as a core element plays a decisive role in determining the electrochemical performance of LSBs. As for electroactive organosulfur polymers, their sulfur content, LiPS trapping ability, and structural stability still need to be further promoted through molecular engineering of polysulfide chain length, functional linkers, polar groups, heteroatom doping, conjugation effect, and topological structures. S‐cPAN may be the most promising sulfur‐containing polymer for successful application, yet the understanding on its exact molecular structure and reaction mechanism as well as the influence of electrolyte still require further study. As such, in situ characterization techniques and theoretical investigation could be integrated to offer in‐depth understanding of battery chemistry by monitoring the structure and morphology evolution of organosulfur polymers during discharge/charge process. In addition, developing controllable, low‐cost, large‐scale synthesis methods should also be considered. As for polymer‐based carriers for sulfur, future work could be concentrated on molecular engineering of polymers and construction of multicomponent nanostructures to strengthen LiPS trapping, enhance kinetics, and promote sulfur content. Meanwhile, the incorporation of conductive carbon in polymer‐based carriers may be needed to promote electronic conductivity of sulfur cathode. Second, promoting electrochemical performance also depends on the binders and separators in addition to sulfur cathodes. Appropriate modifications on conventional binders (e.g., introducing polar groups and increasing adhesion) and multifunctional separators (e.g., regulating pore size and incorporating polar groups), exploring well‐designed polymer barrier layers between the cathode and separator are also expected to effectively suppress the shuttle effect. Finally, developing polymer electrolytes with high ionic conductivity, superior mechanical/chemical stability, and good interface contact is viewed to be the most promising route to completely overcoming the dissolution of polysulfides.

Besides, based on their similar physicochemical properties of sodium, potassium, and lithium and hence the analogous working principles, the polymers employed in LSBs include cathode, binders, separators, and polymer electrolytes are theoretically used in sodium–sulfur (Na–S) and potassium–sulfur (K–S) battery systems. More importantly, the abundant reserves and low cost of Na and K make Na–S and K–S batteries can be considered as promising alternatives to LSBs. However, the same problems such as shuttle effect, electrical insulation of sulfur, and discharge products for LSBs also exist in Na–S and K–S batteries, and are even greater. In addition, the larger ionic radius of K^+^ and Na^+^ than Li^+^, and higher redox potentials may lead to different in working voltages, capacities, and redox kinetics. The larger volume change upon the full sodiation (171%) and potassiation (296%) would result in the serious structural collapse. Besides, the higher activity of Na and K than Li may bring about the unstable and fragile solid electrolyte interphase (SEI), leading to lower Coulombic efficiency and higher polarization. In terms of such challenges, future explorations in Na–S and K–S batteries are highly required in designing functional polymers particularly for polymer cathodes. Anyway, polymers with structural and functional diversities would broaden the scope of LSBs and drive the future development of other metal–sulfur batteries.

## Conflict of Interest

The authors declare no conflict of interest.
